# Supplementary material for: Meta-Analysis and Topological Perturbation in Interactomic Network for Antiopioid Addiction Drug Repurposing
Source: J Chem Inf Model. 2025 Nov 4;65(22):12313–30. doi: 10.1021/acs.jcim.5c02263 (PMC12648648; doi:10.1021/acs.jcim.5c02263)
Supplement: Supplementary file 1 [file ci5c02263_si_001.pdf]

Supporting Information: Meta-analysis and Topological Perturbation in Interactomic Network for Anti-opioid Addiction Drug Repurposing

Chunhuan Zhang[1], Sean Cottrell[2], Benjamin Jones[2], Yueying Zhu[1], Huahai Qiu[1], Bengong Zhang[1], Tianshou Zhou[3], and Jian Jiang[1,2*]
[1]Research Center of Nonlinear Science, School of Mathematics and Statistics, Wuhan Textile University, Wuhan, 430200, P R. China
[2]Department of Mathematics, Michigan State University, East Lansing, Michigan 48824, USA
[3]Key Laboratory of Computational Mathematics, Guangdong Province, and School of Mathematics, Sun Yat-sen University, Guangzhou, 510006, P R. China

October 10, 2025

S1 Gene significance ranking of seven datasets

This study uses a topological property evaluation method based on multi-scale analysis to quantify the importance of each node in protein interaction networks. We first identified top 300 key genes by this method from each dateset, and then collected these key genes from seven datasets to form the final key gene set with 1865 ones after the elimination of redundancy. The specific information of top 300 key genes identified in each dataset is summarized in Table S1.

Table S1: The details of top 300 key genes identified from seven datasets

GSE174409	GSE182321	GSE194368	GSE210206	GSE210682	GSE260711	GSE167922
IL6	FN1	IL6	ALB	HOXB5	RPS27A	ACTB
IL1B	CD44	IL1B	FABP1	HOXA5	RPS3	GRIA1
IFNG	CDH1	VEGFA	DNAI1	PAX2	RPL7A	TAF1
TLR2	CXCR4	CD44	ROPN1L	FOXG1	FOS	RBBP8
CCL2	COL1A1	TLR2	PLG	HOXD4	RPS3A	GRIN2A
FPR1	APOB	COL1A1	CAV1	HOXB4	RPL30	XRN1
OSM	SPP1	CCL2	AGR2	HOXB7	RPSA	RB1
FCGR2A	LUM	MYC	TFF3	HOXC4	RPL4	DLG1
SELP	COL2A1	TIMP1	SPAG6	SLC17A7	RPLP1	SIRT1
FCGR1A	VCAM1	SERPINE1	APOA4	TBR1	EEF1A1	PHF20
TREM1	CCL19	THBS1	RSPH4A	HOXC5	RPL7	FOXK1
SIGLEC7	CSF3	CD163	AHSG	HOXB6	RPS15A	QPCTL

*Corresponding author. Email: jjiang@wtu.edu.cn

CXCR2	SERPINE1	PLAUR	DNAH5	NTS	RPL10A	ADCY8
CSF3	CCRL2	IL2RA	TTR	HNF4A	RPL14	REV3L
SOCS3	CCL20	SOCS3	LCN2	HOXA7	RPS8	ACTA1
SELE	MMP14	CXCR2	HYDIN	HOXA6	RPS23	NUP98
EDN1	COL6A2	F3	CLDN3	TYR	RPS12	TBX5
FPR2	PRF1	LOX	CCNO	FOXD3	RPS10	WDR20
SERPINA1	TWIST1	CDKN1A	NR1H4	MITF	RPS7	POU3F1
CXCR1	CXCL1	SERPINA1	ZMYND10	IGF1	EEF1B2	ZYX
CCL20	CCL21	LIF	GCG	FEZF2	SOX2	MYO5A
TTR	KLF4	FCGR2A	SERPINC1	TTR	RPS6	MON2
CXCL11	CFH	OSM	ENKUR	EMX2	RPS9	NEUROD1
CD93	CXCL2	CD14	MMP1	CHRM1	RPL10	TRUB1
HAMP	HBA1	ACTA1	KRT20	SIX6	RPL12	APBA3
FFAR2	PGR	WNT3A	TEKT1	SLC22A8	RPS4X	GRIK5
ITGB3	COL3A1	VDR	DNAAF1	TYRP1	RPL19	HAPLN1
THBD	PCOLCE	ADM	A2M	CYP11A1	RPL26	MAP6
CR2	SELE	TGFBI	MUC13	RPE65	RPL28	GRIN3B
THBS1	CXCL11	CR2	KRT5	HBE1	RPS29	TRPC3
IL1R2	ITIH2	UNC45B	SPAG1	HOXD9	RPL32	TNRC6C
CDKN1A	CXCR6	CCL20	RSPH1	TRPV4	TCP1	ZRANB3
CD207	OGN	HAMP	DNAAF3	TFAP2A	RPL36A	RD3
CCL8	PAX1	S100A8	DAW1	HOXA3	EIF3E	LHX3
GADD45B	EPHA2	S100A9	DNAH11	ODAM	MRPL11	PAK3
CP	COL6A3	HLA-G	NR1I2	SLC18A3	EEF1D	IMPA1
NGFR	KRT19	CISH	AQP4	NKX6-1	STX5	SYN2
S100A8	PLAU	SLC11A1	MCIDAS	HSD17B2	HNRNPA1	ANO6
IL2RA	SERPINH1	ZC3H12A	SERPINA1	HOXD3	RPL222L1	ANO2
IFNA5	MSX1	ITGA5	CLDN5	KDR	SURF1	GREB1
IL4R	SLC47A1	COL4A1	DRC1	LBX1	PTBP1	TAF1L
LIF	LAMC2	TNFRSF8	EFHC2	HOXA4	NOC4L	SLC8A1
SLC11A1	KLRB1	FPR1	MORN5	HOXD8	EPRS	PALB2
CD14	LYVE1	SERPINH1	TYR	SLC10A2	MRPL16	SPICE1
ANGPTL4	LEPR	SOCS1	CP	PMP2	BDNF	MAP1B
CLEC4D	FZD2	PGF	SERPINE1	OGN	COL1A2	APBBB2
CDK2	ADAMTS1	THBD	CLDN2	SLC5A7	COPG2	NBN
GPR84	RUNX3	CALCA	ABCC3	EMX1	SRP54	ZFP36
SERPINA3	NPC1L1	BATF	CLCA1	TRPM1	GOLGA2	SOX11
IL1RL1	EYA1	GADD45B	EPS8L3	HOXB2	UGP2	SMG1
ADM	MYOC	FABP4	NME5	LCP2	OSTC	GABRG3
FOSL1	C6	CD93	CDH17	AGTR1	SRSF6	GNPDA2
LRG1	SIX2	CP	TRPV4	AGTR2	PFDN6	LINGO2
S100A9	HBE1	PIM1	ANXA1	TLX1	GADD45B	IFI444L
ZFP36	DSP	TNFRSF12A	REG4	MAG	NES	RSAD2
CD209	ATF3	CCL8	DCT	ANKRD666	YKT6	ADAMTSL1
SIGLEC9	SOST	ANGPTL4	SPEF2	OTP	SLC25A5	SBSPON
RGS1	FOXC2	FOSL1	MUC16	SLC30A3	PGM1	NUP153
SI	SFRP2	PAPPA	VIL1	NEUROD6	LMNA	MIOX
MT1X	KRT17	GADD45A	SI	NR5A2	PSMC4	RBPJL

CISH	VIM	ANXA8	UGT2A3	TMC5	ANXA5	BDP1
OSMR	KLF2	FGF16	CLDN16	HOXB3	DDX18	TRPA1
IL18RAP	CETP	ZFP36	MUC17	CLIC6	MT-CO1	SMADCAD1
SFN	ITGBL1	TNFSF14	RPE65	UGT2A3	OTX2	NUDT8
TNFRSF10C	PCSK9	CEBPD	AQP1	PPP1R1B	COG6	CDH18
HBG2	MARCO	IL18RAP	TM4SF4	AMBN	DDX24	ZBTB39
STC1	PGF	IL1R2	NR0B2	SOSTDC1	GOLGA5	FAM83F
MAFF	ZIC2	IL18R1	FOXJ1	CYP26C1	ILK	SNTG1
MYO1G	GPC3	ADAMTS9	AGXT	CLCNKA	PHF21A	CEP135
CHI3L1	CABP4	LILRB3	FRK	WIF1	IRF3	AIPL1
ANKRD222	NR4A1	C8B	LTF	UGT2A2	GADD45G	SOS2
ADGRE1	SERPIND1	KCNN4	MITF	SLC6A5	LDHA	JMJD1C
CASP5	PTPN222	MYO1G	IQUB	SCN5A	GORASP2	LRTM2
BCL2A1	SLC26A2	CD160	ARMC3	PAX8	DDIT3	MTMR7
RAC2	ZPBP	STEAP4	CEACAM5	SLC38A11	EFNB2	PLCXD2
CALCA	HLA-DQA1	GBP2	PTGDS	HOXB8	MSX1	NPAS3
TM4SF1	ACP5	VSIG4	CLDN4	DKK2	ACTN1	HERC3
MT1M	SPINK13	CHI3L1	TYRP1	NKX6-3	SYMPK	HOXD9
TNFRSF10D	GZMM	TM4SF1	GFAP	SLCO1B3-SLCO1B7	COL2A1	HCN3
AHSP	IL9	GPR182	MMP7	SLCO1B3	FZD1	SYTL3
MT1A	ALX4	RIPK4	FAM216B	PHOX2B	ABAT	LDLRAD2
FCRLA	GPR25	ADAMTSL4	LAMB3	GC	FGFR1	SSC5D
CD300E	TACSTD2	OSMR	SULT2A1	GREM1	PCOLCE	PEX3
RGS16	SLC22A6	STAB1	TGM2	GDF3	MT-CYB	ACBD4
MYBPH	CEP555	CCL26	C4BPA	EPAS1	ING3	ACSM1
CLECLEC4F	PCK1	TFPI2	OTX2	SLC39A12	SURF6	SELP
ADGRG3	PRRX2	PDPN	PIGR	CRYM	COX17	ERCC6
F2RL3	PROK1	C1R	TM4SF5	GPNMB	TLE4	DOCK4
STC2	COL8A2	MFAP2	ST6GALNAC2	MAFA	LMX1B	NSUN5
VTCN1	HBD	MYBPC2	F5	BCAN	MOGS	JADE3
SLC9A4	HBB	LOXL4	FABP2	MEP1A	B2M	ANKRD1
CDC20B	CYBRD1	RIPPLY3	SERPINB2	FCER1A	SPARC	HES4
EMP1	PTGDS	ACTG2	ALDOB	LRRC71	TAF10	SACS
CPB1	SLC22A1	SLAMF8	TIMP3	HBD	GNB4	GALNTL6
THRSP	CD209	KISS1	PTGER2	LAMB1	CETN2	ADTRP
ALLC	LYZ	ALOX15B	VIP	TFAP2C	PPAT	EPPK1
SLX1A	ABI3BP	BCL2A1	ANKRD66	ABCA4	S100A10	NFASC
BOLA2B	PRDM6	SFN	SLC2A7	TLX1NB	PDHX	USP35
GLYAT	FBXO39	SPHK1	DNAH7	SKOR2	ABCF1	PRSS48
HILPDA	FOXD1	NLRP6	CEACAM6	PHOX2A	FZD3	ZNF121
GPRC5A	OMD	SLC39A14	OLFM4	OAS2	GOLGA4	E4F1
MPZL2	SLC2A3	STC2	PKP1	GBP1	HLTF	BBX
APOLD1	PRG4	SLC16A3	AQP3	TIMD4	NFIC	MSTN
YBX3	AGTR1	ANKRD1	IQCD	SERPINF1	VP53	CCNI
SLC2A5	KLHDC7A	PRKAG3	MAOB	GHSR	ACADVL	LAYN
IL4I1	OLFML2A	SLC17A3	AK8	UTS2	DPM1	SOCS1
TSSK1B	ITK	SLC22A11	KRT17	TLX3	SLIT2	MANEA

NPAS4	OSM	SIGLEC7	RIBC1	ARHGEF5	S100A13	RUNX1T1
ADGRE2	MFAP2	TEAD4	ADH1C	C1orf141	HSPB1	BCL3
GJB3	SEMA3C	MYBPH	C1R	DNAJC5G	BMP7	LINC02043
SLAMF8	ECM2	TYMP	AMPD3	SLA	DBI	LDLRAD4
STX11	C7	MAGED4	CLDN19	HMGCS2	MBNL1	MPPED1
DPEP3	EFNA4	MAGED4B	ACTG2	PRR27	PSENEN	HIVEP1
PLEKHS1	SPINK9	POTEG	SERPINF1	FRK	FOXA1	HOXB5
CTAGE16P	TFPI2	ARID5A	FOLR1	SLC2A5	SSR2	FBXO34-AS1
RPS2P1	THSD4	KLHL40	FBN1	SLC13A4	RAB12	LINC02958
SLC1A7	APOBEC3B	IL27	ITGB4	SLC6A20	DPAGT1	LIMASI
LINC01337	APOBEC3C	ST14	GALNT5	KCNE4	MRPL555	REPIN1-AS1
SLC22A2	LMX1B	DES	HMGCS2	COBLL1	LMX1A	RBM12B-DT
MRPL40P1	IZUMO1	GPR139	LRRIQ1	MYH2	ZCCHC17	LINC02977
ZNF628-DT	ZP3	ANKRD22	CLDN1	SLN	ZNF598	STIM1-AS1
ADAMTS9	SPINK2	GRHL3	SLC22A8	C5orf58	BTG2	RNF213-AS1
LINC02605	DSG2	NFIL3	CATIP	LMOD2	DPM3	LINC00987
STEAP4	UPK1B	MMP19	FCGBP	XIRP2	PUSL1	LINC02809
DEPP1	UPK3B	BACE2	CLIC6	FCRL3	MSI1	LINC01589
VN2R3P	HGFAC	CATSPER1	CAPSL	POU2AF1	ROMO1	HIF1A-AS1
VN1R40P	BNC2	MALL	TRIM29	SCGB2A1	PPT1	TMEM265
NMRK2	CDC45	GPRC5A	TEKT4	ANKRD30A	ODF2	PANDAR
OR10G8	FGL2	GABRP	MYH14	GBX1	COL6A2	LANCL1-AS1
PDE4DIPP4	MIA	EMP1	ANKUB1	DBX2	UGDH	MIR12136
STIP1P3	P2RY2	PLAC1	SPTLC3	DMRTA2	PRPF4B	DNASE1L2
LINC00558	TP63	C2CD4A	AGR3	MAB21L2	GYG1	FGF14
FAR2P1	AJUBA	POTEH	GPA33	VSX1	MT-ATP8	MLANA
MARK2P16	GSTM1	SLC34A2	HOXB9	FOXB2	KRT18	PTP4A3
LINC02574	SCNN1A	NPAS4	CD274	SLC13A2	NDUFAF3	CELF3
BEND3P1	SEMA3E	MPZL2	FXYD3	HOXD1	ANP32E	COBLL1
CCDC188	SLC5A9	HES7	SLC5A5	KCNJ13	GOSR2	SENP7
FAM66E	TRABD2B	LMX1A	GMPR	HPGD	CSPR2	PNMA8B
OSMR-DT	CSTA	DKKL1	TM4SF20	HMX2	LGALS1	GPAM
MEDAG	SLC22A8	ITPKC	DMBT1	BARX2	NAPA	BPESC1
C11orf91	SMCO2	KCNJ15	PTHLH	RASSF6	SLC39A7	KIAA0408
SSBP3-AS1	CDRT15	GPR84	ACE2	MGP	PEX6	ZFYVE16
SNORD3B-1	HFE	CH25H	FAM81B	STX11	CLU	NUP58
TMEM92-AS1	PTGDR	ULBP1	GJB2	SPINK6	TXNIP	YPEL1
DLX4	KCNJ13	MYOM3	HKDC1	HOXB-AS3	GULP1	AADAT
APOL4	CACNA2D4	CTAGE14P	CXCL14	HOXA-AS3	ID3	PGPEP1
RAB20	DGCR6	MGAT4EP	ANXA4	LOC105378036	TSPO	TOR4A
CRIPTO	CTSW	GSTA9P	SLC6A20	MSGN1	ARID3A	PARPBP
C11orf96	HCAR3	FER1L6-AS1	RHOD	LOC105370849	MEST	SLC52A1
WNT8B	HCAR1	RPL13AP20	DCDC2	LOC105370432	NBL1	RANBP17
TRPC6P1	SLC9B1	LINC00572	CCDC170	LOC105369823	RNF13	CDK15
S100A3	MFSD2B	FAM225B	SLC40A1	PGR-AS1	GPR107	AK5
THBS1-IT1	ANKRD20A2	PSPC1P1	GBP2	LOC105377356	CALB2	SNORD4A
UNC93B3	ARHGEF5	LINC01502	CXCL17	LINC02663	NRP2	RSPH14
RNU1-88P	TCF23	NEAT1	RORC	LINC02070	JMJD6	MICALL2

HILPDA-AS1	CIB4	NIBAN1	PDE11A	LINC01594	XPOT	FAM110D
SNHG27	ACADL	MIR155HG	GALNT14	LOC105370994	SPRY1	TNIP3
SLC25A28-DT	IFNL2	LINC02457	SPINK1	DYNLT5	SART1	PLPPR3
RNU1-30P	SLC16A9	SECTM1	TRPM1	LINC01551	ZFPL1	BICC1
SNORD14E	SOX7	LINC02791	TTC29	LINC00261	PLTP	EGFL8
SECTM1	UTS2B	FOXL3	IL1R1	ACP3	NSUN6	TRPT1
COX20P1	KLF5	RNF122	GALNT10	CERNA2	FOSB	LOXL4
BAALC-AS1	FAM106A	CHI3L2	RBP2	PNOC	ANP32A	GHDC
MT1L	GRHL3	MT1JP	LGALS4	LOC105370366	NR4A1	AJAP1
GOLM2P1	FOXD2	MTND1P23	SPAG8	LOC105371891	RFX3	SNHG12
MT1JP	EGFLAM	TCERG1P2	ABCA4	UGT2A1	CKAP4	SHISAL1
FCGR1CP	C6orf118	LINC02507	TSPAN8	FAM230J	NSUN5	KIAA1656
LINC01837	SAMD11	MOGAT2	DNAH12	LINC02944	FTL	ZNF518B
KIF18B-DT	CCDC154	RN7SL128P	AK7	MAS1	RSPH9	GFM1
CLEC17A	TTLL2	PKD1P2	TMEM72	CLRN2	ACAP1	MIDN
TBC1D3D	C22orf39	ARSDP1	SCIN	LOC105369333	CSTF3	DERL3
ARL2BPP8	TRIM73	LINC01471	IGFBP7	LOC107984173	CLGN	LRP5L
ZNF80	C21orf58	LINC01554	GGT6	LOC107984611	ID2	ZNF184
LINC02642	SERINC2	RAB20	VWA3A	LOC105379330	PLOD3	ZNF205
LINC02540	CLIC6	PDE4DIPP4	ANGPTL4	LOC105370752	SFRP1	H2BC13
OR5B2	TTC29	BCO1	PRSS3	LOC107985460	POSTN	H3C4
SYNE4	FCN1	ANKRD26P3	HSD17B2	LOC101929633	MPHOSPH8	MCF2L2
MT1XP1	AKR1B10	LINC00322	RASSF6	LOC107986746	ZNF274	LRCH1
RNU1-89P	PGM5	LINC01679	ACSL5	C11orf97	SIRT2	WDR7
GOLGA6A	ESPNL	CXADRP3	TMEM45B	RAMP3	SPTY2D1	ARHGAP45
GOLGA8Q	CCDC81	LCMT1-AS2	MYH2	CFAP74	PHYH	BRD7P3
LINC02970	LEFTY2	ALOX15P2	STOML3	CPB1	PIBF1	PREPL
LINC00996	CLEC1A	ANKRD20A9P	SPEF1	MS4A6E	SNCG	MTFR2
MIR5690	SLC5A7	AMBN	SERPINA4	HOTAIRM1	TPBG	MGC4859
YBX3P1	TBC1D7	TMEM114	SLC24A4	LOC100129455	ARF4	SH3D21
C2CD4A	EVC2	HES2	TSPAN1	GALNT5	NAPRT	MMP21
OR56B2P	CHST4	CPA6	KRT15	KIAA0040	HLA-B	CPXM2
METTL5P1	MLPH	CD164L2	GPNMB	HOXD-AS2	CYB5R1	KRBA2
MITA1	PDZK1IP1	LINC02014	IYD	PLA2G5	STX4	ZNF648
LINC02652	LRRC3	CHIAP3	MAP3K19	SLC16A12	H1F0	CST9
CRISPLD2	GBP2	MTATP8P2	TMEM176B	LOC105377547	UPF2	M1AP
LGR6	MRGPRF	PSLNR	SRD5A2	FAM230G	FXR1	TYRO3P
HIF1A-AS3	ABCC6	LINC02827	RAB11FIP1	LOC107986775	HLA-C	FARP2
ZNF878	SPTLC3	LINC01348	CPM	LOC644936	TRAPPC6A	ABCB6
WDR87BP	LRRC37A2	LINC02279	SGMS2	LINC01366	LONP1	SEMA3C
PRPF39-DT	DPEP2	HMGB3P1	PIH1D2	LINC02303	AURKAIP1	CGRRF1
BTNL12P	SLC6A20	LINC02029	AOC1	LAMP5	ARC	UTS2
SELENOTP2	GRAPL	ASH2LP3	MSX1	LOC107987394	CHD7	SETD9
HMGB1P49	CHODL	TNFRSF10D	DYNLRB2	LOC105377548	DDIT4	RAET1E
HERC2P6	TMEM30B	BFSP2-AS1	CCL18	CGNL1	ZC3H6	OR6B1
OR10V1	LINC01638	DUSP5-DT	FNDC1	LOC105371908	EMC2	DDX11L2
CERNA2	TUBB1	ELDR	ERMN	PLA1A	RBM3	FAM83A
LINC03126	FAM216B	TKTL1	CDC20B	BTG4	MIF	ARHGAP27P1

TMEM114	C1orf185	VASN	HOGA1	ISM2	MSI2	PROSER3
LINC03032	CCN1	CTAGE6	SGPP2	LOC107986669	HM13	GARIN4
RPL5P16	CYP2W1	PLEKHS1	ASGR2	C1orf87	ZFP36L2	SHOC1
FAM225B	FAM167B	POM121L9P	CLSTN2	VWA5B1	ZC3H8	USP27X-DT
THAP12P5	BCL6B	PDE4DIPP7	SPATA17	GZMA	YES1	ANKFN1
ALOX15P2	TPSB2	LINC01202	LGALS3BP	LOC105378598	CKB	RETREG3
PRSS56	KANK2	LINC01880	RGS22	STMND1	RCOR2	ZNF296
LINC01068	SMIM32	LINC01303	SLC2A5	CIBAR2	LIMS1	OXER1
LINC01046	PODN	TCAF2C	IQCG	LINC00698	PAPSS1	SCAND3
KCNIP1-OT1	C8orf88	MEDAG	FANK1	CCDC170	PNRC1	OSBPL7
OR52U1P	RASSF10	C1RL-AS1	HSPB6	PCDHB1	FOXRED1	ROR1-AS1
SNED1-AS1	WFIKKN2	TSIX	TRADD	LOC105378714	LMO3	MRTFA-AS1
LINC02506	GRIN3B	MTCO1P40	SLC44A4	TRIM54	GAS6	LINC01793
SLC2A9-AS1	FREM2	LINC02832	SLC28A3	LOC105377048	RGS3	LINC01524
OR56B1	NPW	PTGIR	GPRC5A	LOC107986154	MAD1L1	SMARCAD1-DT
PPDPFP1	C20orf141	MYEOV	SPATA18	FOLR1	NMT1	LINC02044
LINC01471	MCTP2	LNCOG	ADAMTSL3	SNHG26	COL9A2	TAS2R19
LINC00557	FAM237A	CYP2C23P	BHLHE41	MIR34C	TYRO3	SSX8P
LINC02691	RSPH10B	MIR3149	IL1RAP	LINC02192	ATXN7L3	C1RL-AS1
ACCSL	CAVIN1	MTND4LP30	EPAS1	LOC101927690	SAP30	TRNI
ACSM5P1	C20orf96	YBX3	PPP1R42	AMELY	C9orf72	TRNS1
PRSS21	CH25H	MTCO2P12	SOD3	LMNTD1	PTN	GPR4
REM1	CCN5	IL6R-AS1	EVPL	LOC105379314	THOC3	H2AC7
OR52V1P	CWH43	LINC02786	SULT1B1	LOC100128059	AP2B1	RGS9
RPS7P6	DCDC2B	TMEM217	AKAP14	CDKN2B-AS1	TMEM38B	SLC22A13
PRDM7	CCDC84	HLA-W	MALRD1	SIM1-AS1	YBX3	TMPRSS11D
SUSD2	LOC102723623	RDH10-AS1	TPPP3	LOC105373627	DLK1	RNF6
SIGLEC14	GPHA2	DEPP1	BBOX1	FOXD3-AS1	CCS	LINC00685
RPS3AP32	FBXW10	HSPA7	TRPM3	STING1	IGF2BP1	SLC26A11
BNIP3P16	GIPR	IGHG4	BTC	HOXB-AS1	FAM207A	LEMD1-AS1
OR2AT4	FMO1	HIF1A-AS3	TMPRSS4	MIR31HG	CREB3	BEND2
ISCA2P1	KLK1	SLC35F3-AS1	KCNA5	MTUS1-DT	POLE3	CHODL
BFSP2-AS1	HSPA6	LHX4	RARRES1	ACCS	IGFBP5	SUPT20HL2
DENND6A-DT	C16orf89	RPS2P1	AADAC	LOC107984363	MAFB	EMX2OS
GPR179	CAPN6	PRPH	ISX	LINC02821	C3orf58	MTVR2
SLC13A4	LOC107987290	CASZ1	HSPB8	CRPPA	TRPS1	LINC00957
LINC02864	H4C11	MT1M	DMGDH	LOC105373628	ALDH9A1	PIM1
MIR106AHG	LOC100996747	PDLIM4	BPIFB1	LINC01982	ZRSR2	PSPN
MIR549A	COX16	LINC01968	GBP3	DRGX	PAWR	SNORD121B
BFAR	LRRN4CL	UBL4B	CTSO	LOC339298	MPC1	GLIS2-AS1
RFX6	LOC107986244	TMPRSS13	COLEC12	TFF3	CDO1	RUNDC3A-AS1
TNXA	LOC107984026	LINC01620	WDR38	LOC105371257	DDR1	LINC00486
LINC00851	PKHD1L1	LINC01280	EFHB	NMRK2	ARID5B	C5orf60
SOCS3-DT	LOC105370092	RRAD	TTLL10	FCN3	C16orf87	DPY19L4
LINC01685	FBXO43	SCNN1A	MYOF	LAMP5-AS1	PLP2	BCORP1

RN7SKP170	TENT5A	LINC01666	ACOT11	FAM230A	SIKE1	LINC02370
MIR4422HG	TNIP3	PELATON	PRLR	PAX8-AS1	CD99	IHO1
SUMO2P20	ADGRF2	LHX5-AS1	P2RY10	LOC107986089	SON	HEPHL1
LINC02352	MRLN	DUOXA1	LRRC46	MIR615	LGI2	SERINC2
PGC	LOC107987098	LINC02096	TTC16	LOC105373968	A1BG	RTN4RL2
GUSBP12	CFAP97D1	MTND4P12	SLC16A10	LINC01116	PHF23	FAM111B
IL17REL	KLRG2	TBILA	FAM3D	LOC107984904	PHLDB1	SFT2D2
LINC02343	ASIC5	UICLM	C12orf71	MIR34B	LRRC42	TOPAZ1
MRPL45	ACY1	LINC02032	HOXB8	NPBWR1	CYB561D2	SPATA41
LINC00207	CNPY1	MTCO1P12	COPZ2	LOC105375977	NPRL2	EFCAB8
LINC02786	CEMIP	OTOP1	SLC39A12	LOC100128276	CRABP2	RESP18
SH2D2A	CCN3	VSTM1	POF1B	TP73-AS3	UVSSA	BEND4
RPL35P5	HSPB9	LY6G6C	PHEX	LOC105373313	NSMCE2	OR4N2
KRT80	CPED1	AURKBP1	TBC1D8B	CDCP1	INSM1	ZNF805
MMP19	MAB21L4	BMPR1AP1	CDKN2B	TIMP4	ANP32B	FAM180B
CCNYL4	NPFFR2	NCBP2L	PIP5K1B	LOC105374568	MPST	LINC02092
MUPP	SLFN12	RAB44	HTR2C	LOC401021	SYNC	LINC02898
LINC01348	FAM189B	RPS14P8	CCKAR	LOC105373754	DEDD2	MIRLET7F1
KCNE4	SMYD1	ADGRF4	RASSF9	HR	RRBP1	KRTAP5-7
RNF122	LRRC74B	LINC01016	PMCH	SMUG1-AS1	MSANTD4	ZNF705A
LINC01554	SLA2	INTS9-AS1	ISM2	LOC100240735	TMEM107	LINC02072
DPPA3P11	CRABP2	TREML3P	SAMD15	LOC105379505	FANCF	CLEC4GP1
ABCF2P1	CXorf40B	YBX3P1	FILIP1L	LOC107986865	MAGEH1	PRDM16-DT
FAM136BP	KLHL33	LINC02287	SLC13A4	LINC01346	PPIL6	MIAT
OR8Q1P	PLBD1	FAM230B	SOSTDC1	LOC105378379	CEP95	ODF3B
GFRA4	AOX1	USP30-AS1	RAX	LINC00581	FAM184A	DTX2P1-UPK3BP1-PMS2P11
SLCO4A1-AS1	TEX12	TCAF2P1	MS4A8	LOC105372636	TOX3	MBOAT4
GEMIN8P3	JAML	CPHL1P	HCAR2	MIR1298	RBP4	SNORA21
BTNL3	HPS5	HLA-J	TREM1	SLC39A12-AS1	IGDCC3	RPL31P11
DUSP13B	ZBTB12	LINC02739	BTNL8	LINC02381	SDHAF1	CCER2
MEPE	LOC105371933	RPL35AP21	BTNL3	LINC00305	FJX1	PAFAH1B2P2
LINC02989	DNAH3	LINC01982	TNFRSF13C	LOC102724027	UNCX	RAD21-AS1
FAM83E	TNFAIP8L2-SCNM1	IQCA1L	BLNK	LOC105375138	PDE10A	ACTG1P4
PCMTD1P3	LOC107986453	LINC02092	CPA2	LOC105375830	KLHDC8A	NRIP3-DT
PANX3	UPK3BL2	LINC01389	DCDC1	LOC107985537	MFAP2	LINC02751
RPS14P5	TTC6	MIR5188	ADTRP	LOC107986171	WLS	MIR6835
APOBEC3A	TSPAN10	LINC00423	NWD1	MT1H	PTRHD1	MIR7705
OR8A1	TSPYL6	DLX4	ZG16B	OVOL2	ECEL1	LCMT1-AS1
RNU7-171P	TBC1D29P	APOL4	PRAP1	LOC100240734	ZFHX3	LYPLAL1-AS1
CHIAP3	FAT4	ELOCP18	MYO7B	LOC105378751	SPAG16	CASC23
MIR412	FPR3	MT1A	CCDC146	SFTA1P	GPS2	VEGFB
SCYGR4	GJB4	KIAA0040	SLC4A5	CDHR4	C19orf81	ZNF17

MIR378J	CPZ	RGS16	CAPS	LOC112268220	LRRC4B	MZF1
NOS2P3	DUOX2	TCAF2	CPA4	TMEM72	GSE1	TRND
NDUFV1-DT	HDGFL1	AADACL4	KCNJ13	LARGE2	NKAP	BEND3P3
RPL36AP14	OVCH1	GOLGA6C	AIFM3	LINC01085	IFT27	TBC1D3P1-DHX40P1
ADGRE4P	CPXM1	MIR549A	CCDC33	TFAP2A-AS1	HS2ST1	SNORA13
OR52P1	CA5A	SCAT1	CCDC178	EMX2OS	PLEKHA5	SNORA2C
DUSP16	TRPC7	APOBEC3A	PRND	LOC101928722	C8orf33	SNORA25
HSFX2	AXDND1	MATN4	SCGN	FMO3	MYADM	SNORA57
PGGT1BP1	PPDPFL	POM121B	SLC17A8	LOC105374107	PHLDA1	LRBA
GLTPD2	LOC107987237	ZSCAN10	NPHP1	LOC105370986	EBF1	CLEC19A

S2 Datasets for machine learning

To construct a predictive model for target binding affinity, this study first used key genes identified from seven datasets to collect 72 inhibitor related datasets in the CHEMBL database (the detailed information can be found in Table S2). In the training of machine learning model, we take the SMILES sequences of each compound as input features, and their corresponding binding affinities (in kcal/mol) as target variables. Subsequently, we used the model to predict the target affinity of approved and investigational drug molecules from the DrugBank database.

Table S2: The ID name and sample size of 72 inhibitor datasets

CHEMBL ID	data size	CHEMBL ID	data size	CHEMBL ID	data size
CHEMBL216	1889	CHEMBL3100	301	CHEMBL4691	684
CHEMBL1801	974	CHEMBL3106	194	CHEMBL4767	217
CHEMBL1836	753	CHEMBL3119	473	CHEMBL4789	450
CHEMBL1856	403	CHEMBL3194	187	CHEMBL4835	503
CHEMBL1995	260	CHEMBL3286	1083	CHEMBL5247	162
CHEMBL2047	327	CHEMBL3359	291	CHEMBL5314	547
CHEMBL2068	549	CHEMBL3401	208	CHEMBL5319	238
CHEMBL2073	149	CHEMBL3475	260	CHEMBL5378	130
CHEMBL2083	347	CHEMBL3572	1288	CHEMBL5398	25
CHEMBL2085	331	CHEMBL3650	2960	CHEMBL5480	259
CHEMBL2107	895	CHEMBL3785	313	CHEMBL5493	180
CHEMBL2147	4601	CHEMBL3869	798	CHEMBL5918	47
CHEMBL2319	237	CHEMBL4029	314	CHEMBL6003	77
CHEMBL2434	1044	CHEMBL4073	552	CHEMBL6007	535
CHEMBL2525	1382	CHEMBL4076	360	CHEMBL1250348	261
CHEMBL2593	353	CHEMBL4223	225	CHEMBL1293255	313
CHEMBL2717	192	CHEMBL4306	732	CHEMBL1293293	273
CHEMBL2730	490	CHEMBL4394	366	CHEMBL1741179	469
CHEMBL2778	289	CHEMBL4409	4520	CHEMBL1741186	2518
CHEMBL2789	671	CHEMBL4427	538	CHEMBL1741200	221
CHEMBL2889	274	CHEMBL4462	911	CHEMBL1741213	805
CHEMBL2959	982	CHEMBL4507	26	CHEMBL2424504	330
CHEMBL3018	465	CHEMBL4607	468	CHEMBL3392948	435
CHEMBL3060	141	CHEMBL4616	1353	CHEMBL3714079	579

S3 Evaluation metrics

To evaluate the performance of the regression model, we used three key metrics: Pearson correlation coefficient (P) and root mean square error (RMSE), and coefficient of determination (R^2). The definitions and formulas for these metrics are as follows: The Pearson correlation coefficient (P) is defined :

$$P(x,y) = \frac{\sum(x_i - \overline{x})(y_i - \overline{y})}{\sqrt{\sum(x_i - \overline{x})^2 \sum(y_i - \overline{y})^2}}, \tag{1}$$

where $\overline{x}$ and $\overline{y}$ represent the mean of vectors $x = (x_1, x_2, ..., x_n)$ and $y = (y_1, y_2, ..., y_n)$, respectively.

The root mean squared error (RMSE) is calculated as:

$$\text{RMSE} = \sqrt{\frac{1}{n}\sum_{i=1}^{n}(y_i - \hat{y}_i)^2}, \tag{2}$$

where y_i is the true value and $\hat{y}_i$ is the predicted value for the i-th sample.

The coefficient of determination (R^2) is calculated as:

$$R^2 = 1 - \frac{SS_{\text{res}}}{SS_{\text{tot}}}, \tag{3}$$

where SS_{res} is the residual sum of squares, which is the sum of the squares of the difference between the actual observed values and the model predicted values, and SS_{tot} is the total sum of squares, which is the sum of the squares of the difference between the actual observed value and the average observed value.

S4 Performance and parameter settings of the model

The hyperparameter configuration details of each machine learning (ML) algorithm are listed in Table S3. In order to ensure the reliability of the prediction results, we compared the performance of different ML models. We chose three different regressors, including support vector machine (SVM), gradient boosting decision tree (GBDT), and random forest (RF), as well as three kinds of molecular reprentations, inclduing sequence-to-sequence autoencoders fingerprints (AE-TP), bidirectional transformer fingerprints (BET-TP) and a traditional 2D fingerprint Extended Connectivity Fingerprint (ECFP). The comparison results indicate that the model integrating SVM algorithm with three molecular fingerprints fused together performs better than other model configurations on most datasets with three evaluation metrics, including Pearson correlation coefficient, root mean square error, and coefficient of determination. The details of predictive results of binding affinites on 72 inhibitor datasets can be found in Tables S4, S5, and S6.

Table S3: Configured hyperparameters of RF, SVM, and GBDT across different training-set sizes. All other parameters are set to their defaults.

Train Size	RF Parameters	GBDT Parameters	SVM Parameters
< 1000	$n\_estimators = 10000$<br>$criterion =' mse'$<br>$max\_depth = 7$<br>$min\_samples\_split = 3$<br>$min\_samples\_leaf = 1$	$n\_estimators = 10000$<br>$max\_depth = 7$<br>$min\_samples\_split = 3$<br>$subsample = 0.7$<br>$learning\_rate = 0.01$<br>$max\_features =' sqrt'$	$C = 10$<br>$gamma =' scale'$<br>$kernel =' rbf'$
$[1000, 5000)$	$n\_estimators = 10000$<br>$criterion =' mse'$<br>$max\_depth = 8$<br>$min\_samples\_split = 4$<br>$min\_samples\_leaf = 2$	$n\_estimators = 10000$<br>$max\_depth = 8$<br>$min\_samples\_split = 4$<br>$subsample = 0.5$<br>$learning\_rate = 0.01$<br>$max\_features =' sqrt'$	$C = 5$<br>$gamma =' scale'$<br>$kernel =' rbf'$
> 5000	$n\_estimators = 10000$<br>$criterion =' mse'$<br>$max\_depth = 9$<br>$min\_samples\_split = 7$<br>$min\_samples\_leaf = 3$	$n\_estimators = 10000$<br>$max\_depth = 9$<br>$min\_samples\_split = 7$<br>$subsample = 0.3$<br>$learning\_rate = 0.01$<br>$max\_features =' sqrt'$	$C = 1$<br>$gamma =' scale'$<br>$kernel =' rbf'$

Table S4: Comparison of root mean square error (RMSE) results among GBDT, SVM, and RF models

Datasets	fingerprint	GBDT	SVM	RF
CHEMBL216	BET	1.071388	0.985931	1.216259
	AE	1.126656	0.998131	1.235929
	ECFP	0.982366	1.061802	1.214033
	AE+BET	1.080866	0.967812	1.203641
	AE+ECFP	0.989746	0.975977	1.176661
	BET+ECFP	0.971925	0.966894	1.173668
	AE+BET+ECFP	0.99932	0.954554	1.172008
CHEMBL1801	BET	1.010658	0.887723	1.161543
	AE	1.180317	0.933347	1.366395
	ECFP	0.887757	1.028009	1.070377
	AE+BET	1.064875	0.882476	1.214211
	AE+ECFP	0.951094	0.918043	1.1268
	BET+ECFP	0.903673	0.89941	1.074171
	AE+BET+ECFP	0.955566	0.882296	1.117009
CHEMBL1836	BET	1.072084	0.976613	1.208642
	AE	1.09886	0.995875	1.189468
	ECFP	1.008336	1.088566	1.211367
	AE+BET	1.072046	0.966309	1.178966
	AE+ECFP	0.997127	0.986408	1.16044
	BET+ECFP	0.992955	0.979093	1.172634
	AE+BET+ECFP	1.007991	0.962115	1.159672
CHEMBL1856	BET	1.119997	1.045734	1.216323
	AE	1.159007	1.075292	1.194121
	ECFP	0.970625	1.154449	1.027618
	AE+BET	1.123476	1.037807	1.179578
	AE+ECFP	1.001626	1.041521	1.049539
	BET+ECFP	0.99023	1.037256	1.062182
	AE+BET+ECFP	1.024382	1.020255	1.081321
CHEMBL1995	BET	0.872893	0.854507	0.887339
	AE	0.859993	0.812533	0.908457
	ECFP	0.882405	0.972627	0.909822
	AE+BET	0.858518	0.821778	0.882989
	AE+ECFP	0.843919	0.853017	0.883287
	BET+ECFP	0.851639	0.873565	0.875626
	AE+BET+ECFP	0.844459	0.83851	0.873429
CHEMBL2047	BET	1.062854	1.055934	1.124383
	AE	1.141658	1.077785	1.155356
	ECFP	1.05842	1.088496	1.051085
	AE+BET	1.089759	1.048285	1.121867
	AE+ECFP	1.047445	1.038755	1.053304
	BET+ECFP	1.024646	1.033218	1.044989
	AE+BET+ECFP	1.042708	1.028503	1.061251

Continued on next page

Continued from previous page

Datasets	fingerprint	GBDT	SVM	RF
CHEMBL2068	BET	0.85033	0.799974	0.88253
	AE	0.911298	0.868622	0.966972
	ECFP	0.788825	0.81715	0.835591
	AE+BET	0.86558	0.807146	0.903475
	AE+ECFP	0.803274	0.803447	0.861931
	BET+ECFP	0.788084	0.778167	0.836937
	AE+BET+ECFP	0.808734	0.785141	0.858067
CHEMBL2073	BET	1.412996	1.256457	1.493268
	AE	1.392021	1.276439	1.464701
	ECFP	1.236983	1.418335	1.342217
	AE+BET	1.373768	1.249289	1.440659
	AE+ECFP	1.247537	1.294265	1.32371
	BET+ECFP	1.282921	1.29716	1.358844
	AE+BET+ECFP	1.286403	1.26729	1.354823
CHEMBL2083	BET	0.836766	0.789192	0.875779
	AE	0.889144	0.802568	0.944111
	ECFP	0.80413	0.846614	0.762011
	AE+BET	0.854384	0.783939	0.895886
	AE+ECFP	0.811497	0.795608	0.812652
	BET+ECFP	0.791853	0.784739	0.784508
	AE+BET+ECFP	0.81136	0.779585	0.821575
CHEMBL2085	BET	0.814553	0.822936	0.831601
	AE	0.84898	0.851767	0.884297
	ECFP	0.818568	0.83386	0.8345
	AE+BET	0.82553	0.82679	0.848382
	AE+ECFP	0.807681	0.818876	0.83797
	BET+ECFP	0.791578	0.803936	0.814908
	AE+BET+ECFP	0.801905	0.809922	0.828261
CHEMBL2107	BET	1.030345	1.00382	1.112101
	AE	1.0779	1.030682	1.136171
	ECFP	0.985586	1.046383	1.076901
	AE+BET	1.038807	0.992951	1.115767
	AE+ECFP	0.976743	0.990417	1.081031
	BET+ECFP	0.966281	0.97749	1.073766
	AE+BET+ECFP	0.981639	0.97323	1.084168
CHEMBL2147	BET	0.935925	0.837762	1.114356
	AE	0.994128	0.840266	1.320574
	ECFP	0.866382	0.973522	1.059414
	AE+BET	0.940214	0.818719	1.153703
	AE+ECFP	0.864352	0.845669	1.101955
	BET+ECFP	0.851806	0.845734	1.039621
	AE+BET+ECFP	0.869984	0.819706	1.074788

Continued on next page

Continued from previous page

Datasets	fingerprint	GBDT	SVM	RF
CHEMBL2319	BET	0.973437	0.928605	0.976333
	AE	1.01127	0.907926	1.037046
	ECFP	0.936684	0.970925	0.994808
	AE+BET	0.981029	0.907652	0.990947
	AE+ECFP	0.939376	0.916195	0.989599
	BET+ECFP	0.931063	0.923372	0.956998
	AE+BET+ECFP	0.942766	0.908852	0.971264
CHEMBL2434	BET	0.944598	0.903994	1.000936
	AE	0.976103	0.893799	1.163223
	ECFP	0.933642	1.028395	0.995262
	AE+BET	0.94427	0.880675	1.042393
	AE+ECFP	0.907154	0.909437	1.018505
	BET+ECFP	0.906155	0.91577	0.973235
	AE+BET+ECFP	0.908359	0.887663	0.996604
CHEMBL2525	BET	0.980745	0.903541	1.051809
	AE	0.994352	0.922704	1.070743
	ECFP	0.917722	0.949609	1.057167
	AE+BET	0.973306	0.894773	1.044305
	AE+ECFP	0.908989	0.893673	1.029757
	BET+ECFP	0.908194	0.884094	1.019914
	AE+BET+ECFP	0.918985	0.879017	1.021676
CHEMBL2593	BET	1.017159	0.974677	1.065457
	AE	1.038732	0.891186	1.063645
	ECFP	0.934511	1.05205	1.009841
	AE+BET	1.011258	0.911879	1.042962
	AE+ECFP	0.932911	0.91729	0.997443
	BET+ECFP	0.930857	0.946985	1.008678
	AE+BET+ECFP	0.945807	0.908471	1.006317
CHEMBL2717	BET	0.969257	0.940496	0.981254
	AE	0.983074	0.937768	1.071531
	ECFP	0.929346	0.954385	0.949842
	AE+BET	0.966128	0.92929	1.00314
	AE+ECFP	0.932141	0.925852	0.978959
	BET+ECFP	0.92882	0.926685	0.941988
	AE+BET+ECFP	0.936329	0.921551	0.965789
CHEMBL2730	BET	0.915403	0.884019	0.970302
	AE	0.931747	0.864947	0.949559
	ECFP	0.923091	1.008547	0.907649
	AE+BET	0.913744	0.858404	0.945588
	AE+ECFP	0.891982	0.887414	0.906184
	BET+ECFP	0.883605	0.893891	0.915113
	AE+BET+ECFP	0.887384	0.865821	0.915544

Continued on next page

Continued from previous page

Datasets	fingerprint	GBDT	SVM	RF
CHEMBL2778	BET	1.033522	0.993317	1.088642
	AE	1.061238	1.000194	1.164263
	ECFP	1.128071	1.218781	1.135867
	AE+BET	1.033154	0.969011	1.108552
	AE+ECFP	1.048773	1.030547	1.107586
	BET+ECFP	1.04055	1.031418	1.078748
	AE+BET+ECFP	1.029204	0.988087	1.087632
CHEMBL2789	BET	0.621521	0.602735	0.692135
	AE	0.635062	0.600307	0.703314
	ECFP	0.554144	0.647372	0.617347
	AE+BET	0.618128	0.588749	0.668097
	AE+ECFP	0.562691	0.580955	0.621434
	BET+ECFP	0.558721	0.581735	0.626398
	AE+BET+ECFP	0.57221	0.572271	0.62759
CHEMBL2889	BET	0.666269	0.640754	0.688806
	AE	0.708159	0.650135	0.72658
	ECFP	0.666018	0.701933	0.649445
	AE+BET	0.680499	0.634324	0.701593
	AE+ECFP	0.65412	0.656	0.662416
	BET+ECFP	0.642191	0.651004	0.649103
	AE+BET+ECFP	0.651874	0.641187	0.66544
CHEMBL2959	BET	0.936328	0.897465	1.017544
	AE	0.963853	0.894799	1.104862
	ECFP	0.869275	0.92202	1.015493
	AE+BET	0.936543	0.876848	1.041933
	AE+ECFP	0.873983	0.86398	1.027906
	BET+ECFP	0.865337	0.867248	0.992574
	AE+BET+ECFP	0.881773	0.857227	1.012276
CHEMBL3018	BET	1.121469	1.017985	1.186011
	AE	1.229802	1.074822	1.352551
	ECFP	1.112034	1.192146	1.194691
	AE+BET	1.15349	1.018508	1.234772
	AE+ECFP	1.101255	1.067067	1.224739
	BET+ECFP	1.058438	1.037915	1.15415
	AE+BET+ECFP	1.087691	1.021996	1.19087
CHEMBL3060	BET	0.769442	0.717171	0.811967
	AE	0.768076	0.725124	0.811093
	ECFP	0.785025	0.788379	0.725542
	AE+BET	0.762389	0.710553	0.802352
	AE+ECFP	0.742344	0.728436	0.739892
	BET+ECFP	0.749692	0.724265	0.74514
	AE+BET+ECFP	0.7438	0.713254	0.75588

Continued on next page

Continued from previous page

Datasets	fingerprint	GBDT	SVM	RF
CHEMBL3100	BET	0.70484	0.702057	0.734347
	AE	0.7399	0.685299	0.834495
	ECFP	0.674024	0.808875	0.711733
	AE+BET	0.714982	0.685349	0.758597
	AE+ECFP	0.677789	0.705011	0.733576
	BET+ECFP	0.665115	0.709921	0.701754
	AE+BET+ECFP	0.679325	0.688487	0.721267
CHEMBL3106	BET	0.856236	0.765781	0.879242
	AE	0.887576	0.741578	0.950464
	ECFP	0.920995	0.902828	0.889208
	AE+BET	0.863823	0.745328	0.899122
	AE+ECFP	0.881455	0.796296	0.901444
	BET+ECFP	0.865433	0.806284	0.856206
	AE+BET+ECFP	0.864057	0.775121	0.878717
CHEMBL3119	BET	1.010732	0.933423	1.04233
	AE	1.016886	0.893622	1.068133
	ECFP	0.91099	1.03001	1.021836
	AE+BET	1.001307	0.895139	1.032482
	AE+ECFP	0.918601	0.907653	1.006894
	BET+ECFP	0.925747	0.923754	1.003171
	AE+BET+ECFP	0.938572	0.893368	1.004065
CHEMBL3194	BET	0.704103	0.689625	0.714919
	AE	0.71201	0.728789	0.739564
	ECFP	0.72715	0.7567	0.718086
	AE+BET	0.701159	0.700709	0.717842
	AE+ECFP	0.698636	0.724749	0.708198
	BET+ECFP	0.699	0.707192	0.696434
	AE+BET+ECFP	0.694566	0.70602	0.701882
CHEMBL3286	BET	0.952386	0.88476	1.065234
	AE	1.010711	0.890415	1.11264
	ECFP	0.849234	0.927454	1.03293
	AE+BET	0.967639	0.866992	1.075047
	AE+ECFP	0.872866	0.858806	1.039629
	BET+ECFP	0.851511	0.851799	1.021446
	AE+BET+ECFP	0.884646	0.84481	1.037089
CHEMBL3359	BET	0.892273	0.837735	0.936237
	AE	0.920441	0.837002	0.952777
	ECFP	0.790794	0.956172	0.821023
	AE+BET	0.890583	0.820625	0.928369
	AE+ECFP	0.810681	0.848387	0.842741
	BET+ECFP	0.807571	0.853968	0.844643
	AE+BET+ECFP	0.826057	0.828194	0.861756

Continued on next page

Continued from previous page

Datasets	fingerprint	GBDT	SVM	RF
CHEMBL3401	BET	0.751011	0.686606	0.792963
	AE	0.772281	0.700845	0.812742
	ECFP	0.812596	0.901901	0.846913
	AE+BET	0.752975	0.683637	0.790429
	AE+ECFP	0.757725	0.74704	0.794044
	BET+ECFP	0.748059	0.738442	0.787557
	AE+BET+ECFP	0.74402	0.707646	0.781418
CHEMBL3475	BET	0.774415	0.721332	0.792873
	AE	0.744277	0.698723	0.783425
	ECFP	0.793224	0.780809	0.781584
	AE+BET	0.750476	0.695954	0.775982
	AE+ECFP	0.740078	0.708426	0.75977
	BET+ECFP	0.75587	0.712562	0.771511
	AE+BET+ECFP	0.741336	0.695833	0.763417
CHEMBL3572	BET	0.836298	0.804033	0.884375
	AE	0.905824	0.802079	0.929336
	ECFP	0.833153	0.875507	0.863038
	AE+BET	0.850584	0.790356	0.888771
	AE+ECFP	0.813515	0.798411	0.861615
	BET+ECFP	0.798095	0.799687	0.849498
	AE+BET+ECFP	0.807944	0.785294	0.857979
CHEMBL3650	BET	0.900853	0.828742	1.046158
	AE	0.941777	0.824473	1.019509
	ECFP	0.810835	0.873444	1.069354
	AE+BET	0.901587	0.807486	1.011821
	AE+ECFP	0.818879	0.804459	1.004195
	BET+ECFP	0.811229	0.806896	1.022508
	AE+BET+ECFP	0.83067	0.793837	1.001859
CHEMBL3785	BET	0.864501	0.798318	0.899298
	AE	0.893529	0.807068	1.009298
	ECFP	0.80922	1.041122	0.861256
	AE+BET	0.869691	0.79126	0.914778
	AE+ECFP	0.814316	0.855709	0.876251
	BET+ECFP	0.808402	0.853732	0.846154
	AE+BET+ECFP	0.822689	0.814872	0.863666
CHEMBL3869	BET	1.15775	1.092857	1.244222
	AE	1.224552	1.067829	1.315626
	ECFP	1.031591	1.174218	1.147229
	AE+BET	1.17455	1.050801	1.267482
	AE+ECFP	1.062733	1.058598	1.198786
	BET+ECFP	1.038379	1.069331	1.168489
	AE+BET+ECFP	1.077276	1.041353	1.203733

Continued on next page

Continued from previous page

Datasets	fingerprint	GBDT	SVM	RF
CHEMBL4029	BET	0.985543	1.0032	1.019152
	AE	1.021831	0.967557	1.032638
	ECFP	1.035755	1.067648	0.992704
	AE+BET	0.995358	0.974576	1.015859
	AE+ECFP	0.975935	0.966779	0.981489
	BET+ECFP	0.965635	0.982305	0.975172
	AE+BET+ECFP	0.966916	0.961196	0.982883
CHEMBL4073	BET	0.973173	0.919991	1.040586
	AE	1.035635	0.935993	1.126844
	ECFP	0.951924	0.963355	0.954157
	AE+BET	0.991491	0.910529	1.063441
	AE+ECFP	0.94432	0.903496	0.988581
	BET+ECFP	0.918627	0.896901	0.950474
	AE+BET+ECFP	0.939794	0.891234	0.987893
CHEMBL4076	BET	0.527072	0.514641	0.54952
	AE	0.526421	0.532875	0.543533
	ECFP	0.545734	0.59767	0.522424
	AE+BET	0.521343	0.515706	0.540225
	AE+ECFP	0.509678	0.527669	0.509696
	BET+ECFP	0.513125	0.520804	0.515973
	AE+BET+ECFP	0.508453	0.511694	0.516518
CHEMBL4223	BET	1.041719	1.072233	1.052793
	AE	1.049059	1.060986	1.06042
	ECFP	0.999941	1.004052	1.012068
	AE+BET	1.034726	1.056534	1.044834
	AE+ECFP	0.991081	1.01234	1.01087
	BET+ECFP	0.99416	1.015148	1.012505
	AE+BET+ECFP	0.998856	1.022143	1.016403
CHEMBL4306	BET	0.627885	0.630851	0.652382
	AE	0.624459	0.636277	0.65454
	ECFP	0.612503	0.631585	0.634753
	AE+BET	0.619446	0.621083	0.644731
	AE+ECFP	0.59475	0.603997	0.630908
	BET+ECFP	0.5982	0.601141	0.628601
	AE+BET+ECFP	0.598312	0.600554	0.630565
CHEMBL4394	BET	1.032077	1.025469	1.063185
	AE	1.035328	1.029505	1.060861
	ECFP	1.015382	1.073062	1.007626
	AE+BET	1.024261	1.013503	1.052294
	AE+ECFP	0.988647	1.010351	1.0081
	BET+ECFP	0.993854	1.012223	1.010565
	AE+BET+ECFP	0.993816	1.001523	1.016979

Continued on next page

Continued from previous page

Datasets	fingerprint	GBDT	SVM	RF
CHEMBL4409	BET	0.994345	0.913443	1.178031
	AE	1.004937	0.894879	1.202121
	ECFP	0.908849	0.986838	1.247055
	AE+BET	0.983623	0.884161	1.174317
	AE+ECFP	0.902634	0.886159	1.17981
	BET+ECFP	0.905208	0.896025	1.180298
	AE+BET+ECFP	0.91785	0.87372	1.167538
CHEMBL4427	BET	0.955709	0.863631	1.0247
	AE	0.981169	0.869099	1.013747
	ECFP	0.883468	1.047245	0.906965
	AE+BET	0.956407	0.846318	0.997287
	AE+ECFP	0.88404	0.902566	0.916612
	BET+ECFP	0.871157	0.904736	0.917073
	AE+BET+ECFP	0.892191	0.869214	0.931119
CHEMBL4462	BET	0.867025	0.778484	1.022065
	AE	0.890418	0.768814	1.028862
	ECFP	0.750183	0.851562	0.89572
	AE+BET	0.863492	0.75731	0.976257
	AE+ECFP	0.762549	0.771152	0.895452
	BET+ECFP	0.762245	0.774032	0.913017
	AE+BET+ECFP	0.783518	0.756234	0.909735
CHEMBL4507	BET	0.675158	0.634263	0.67939
	AE	0.707105	0.690867	0.695081
	ECFP	0.654787	0.671342	0.698816
	AE+BET	0.687008	0.660584	0.683061
	AE+ECFP	0.655148	0.660646	0.674688
	BET+ECFP	0.639339	0.635724	0.665157
	AE+BET+ECFP	0.654492	0.647867	0.668646
CHEMBL4607	BET	1.038807	1.015693	1.075575
	AE	1.093458	1.000434	1.158725
	ECFP	1.05207	1.090219	1.045427
	AE+BET	1.048348	0.982804	1.091313
	AE+ECFP	1.00918	0.995096	1.059269
	BET+ECFP	1.002701	1.011187	1.034403
	AE+BET+ECFP	1.006089	0.982819	1.050904
CHEMBL4616	BET	1.062869	1.043422	1.13844
	AE	1.090795	1.046163	1.208385
	ECFP	1.053843	1.073249	1.17282
	AE+BET	1.060453	1.023689	1.158169
	AE+ECFP	1.02033	1.018173	1.169039
	BET+ECFP	1.014068	1.017834	1.137584
	AE+BET+ECFP	1.019028	1.008153	1.148751

Continued on next page

Continued from previous page

Datasets	fingerprint	GBDT	SVM	RF
CHEMBL4691	BET	0.970869	0.982121	1.004652
	AE	1.025588	0.968098	1.032657
	ECFP	0.967878	1.013407	0.960366
	AE+BET	0.986251	0.958119	1.009162
	AE+ECFP	0.940501	0.937814	0.9635
	BET+ECFP	0.923165	0.942866	0.950652
	AE+BET+ECFP	0.937293	0.931973	0.966317
CHEMBL4767	BET	1.181521	1.1193	1.243706
	AE	1.170412	1.088623	1.197527
	ECFP	1.182571	1.2364	1.168315
	AE+BET	1.162073	1.085286	1.202585
	AE+ECFP	1.10303	1.102001	1.129869
	BET+ECFP	1.114646	1.122726	1.159981
	AE+BET+ECFP	1.109242	1.087527	1.151215
CHEMBL4789	BET	0.901023	0.867166	0.957476
	AE	0.971410	0.890536	1.014620
	ECFP	0.960690	1.019853	0.996392
	AE+BET	0.924047	0.866599	0.971130
	AE+ECFP	0.916806	0.904034	0.972353
	BET+ECFP	0.889983	0.896295	0.949467
	AE+BET+ECFP	0.898607	0.875680	0.955754
CHEMBL4835	BET	0.930986	0.878729	0.997520
	AE	1.006546	0.870472	1.044122
	ECFP	0.862069	0.981532	0.960768
	AE+BET	0.949952	0.857477	0.984816
	AE+ECFP	0.888142	0.877046	0.941001
	BET+ECFP	0.862036	0.875938	0.937599
	AE+BET+ECFP	0.889082	0.855939	0.938642
CHEMBL5247	BET	0.781772	0.736964	0.798038
	AE	0.773397	0.722392	0.803496
	ECFP	0.796401	0.857793	0.805722
	AE+BET	0.769198	0.719925	0.790472
	AE+ECFP	0.764038	0.745717	0.78522
	BET+ECFP	0.76835	0.753874	0.783903
	AE+BET+ECFP	0.761545	0.728017	0.781169
CHEMBL5314	BET	0.890112	0.849342	0.95007
	AE	0.944283	0.8266	1.075997
	ECFP	0.876317	0.983138	0.976918
	AE+BET	0.900401	0.819854	0.981251
	AE+ECFP	0.858039	0.855319	0.967598
	BET+ECFP	0.846129	0.868448	0.927692
	AE+BET+ECFP	0.856326	0.834035	0.944227

Continued on next page

Continued from previous page

Datasets	fingerprint	GBDT	SVM	RF
CHEMBL5319	BET	1.072723	1.084963	1.08944
	AE	1.05291	1.063293	1.056849
	ECFP	1.043352	1.039415	1.009995
	AE+BET	1.055807	1.062401	1.064893
	AE+ECFP	1.014939	1.024558	1.004349
	BET+ECFP	1.025733	1.03189	1.016344
	AE+BET+ECFP	1.024147	1.031974	1.020826
CHEMBL5378	BET	0.9433	0.92669	0.955104
	AE	1.010404	0.946408	0.989342
	ECFP	0.901735	0.931342	0.901396
	AE+BET	0.962855	0.923638	0.962117
	AE+ECFP	0.909244	0.912197	0.917925
	BET+ECFP	0.892574	0.901217	0.907444
	AE+BET+ECFP	0.91154	0.904788	0.922756
CHEMBL5398	BET	0.717459	0.67913	0.734499
	AE	0.689546	0.652949	0.658228
	ECFP	0.686627	0.735886	0.669565
	AE+BET	0.697848	0.664422	0.68511
	AE+ECFP	0.650671	0.670265	0.638226
	BET+ECFP	0.666462	0.681934	0.677592
	AE+BET+ECFP	0.663029	0.666158	0.660159
CHEMBL5480	BET	0.488382	0.512922	0.498292
	AE	0.47755	0.504919	0.487689
	ECFP	0.494391	0.503175	0.488357
	AE+BET	0.478996	0.499878	0.489998
	AE+ECFP	0.473068	0.481257	0.478722
	BET+ECFP	0.477329	0.482175	0.482818
	AE+BET+ECFP	0.472969	0.481283	0.481308
CHEMBL5493	BET	0.860629	0.864694	0.886484
	AE	0.851589	0.820598	0.880594
	ECFP	0.857564	1.008606	0.87728
	AE+BET	0.847975	0.82858	0.86748
	AE+ECFP	0.805598	0.853874	0.837216
	BET+ECFP	0.819864	0.881043	0.85345
	AE+BET+ECFP	0.813743	0.838594	0.843036
CHEMBL5918	BET	0.647477	0.643387	0.658851
	AE	0.641931	0.645185	0.612318
	ECFP	0.661783	0.661149	0.647984
	AE+BET	0.641711	0.641603	0.62942
	AE+ECFP	0.637025	0.641041	0.615541
	BET+ECFP	0.643297	0.640673	0.637439
	AE+BET+ECFP	0.637394	0.638128	0.623311

Continued on next page

Continued from previous page

Datasets	fingerprint	GBDT	SVM	RF
CHEMBL6003	BET	1.308039	1.307777	1.3785
	AE	1.371015	1.334214	1.402608
	ECFP	1.319502	1.402272	1.366721
	AE+BET	1.320013	1.314701	1.367391
	AE+ECFP	1.315201	1.32957	1.355708
	BET+ECFP	1.29891	1.317263	1.348976
	AE+BET+ECFP	1.304083	1.31101	1.348837
CHEMBL6007	BET	0.92147	0.930997	0.940992
	AE	0.998131	0.959299	1.006132
	ECFP	0.94964	0.969271	0.978923
	AE+BET	0.943236	0.934401	0.953997
	AE+ECFP	0.928321	0.924956	0.959801
	BET+ECFP	0.906684	0.912908	0.938381
	AE+BET+ECFP	0.915727	0.914191	0.942308
CHEMBL1250348	BET	0.870198	0.733968	1.037864
	AE	0.862157	0.727149	1.099858
	ECFP	0.75117	0.848671	0.736341
	AE+BET	0.854257	0.717435	1.021087
	AE+ECFP	0.763534	0.750281	0.847196
	BET+ECFP	0.775734	0.75705	0.825671
	AE+BET+ECFP	0.788331	0.731463	0.878087
CHEMBL1293255	BET	0.878313	0.845758	0.920075
	AE	0.888445	0.850746	0.932801
	ECFP	0.895332	0.958988	0.925925
	AE+BET	0.87104	0.834954	0.910553
	AE+ECFP	0.853718	0.863737	0.894447
	BET+ECFP	0.855459	0.862472	0.897098
	AE+BET+ECFP	0.850819	0.842579	0.892046
CHEMBL1293293	BET	0.70295	0.636831	0.736193
	AE	0.697598	0.643176	0.736697
	ECFP	0.648635	0.795107	0.659707
	AE+BET	0.691209	0.626326	0.726426
	AE+ECFP	0.637253	0.675646	0.669632
	BET+ECFP	0.645701	0.673987	0.671885
	AE+BET+ECFP	0.649928	0.646421	0.682333
CHEMBL1741179	BET	0.433355	0.430765	0.43671
	AE	0.438292	0.422733	0.441027
	ECFP	0.454459	0.490716	0.454503
	AE+BET	0.431514	0.420816	0.434697
	AE+ECFP	0.435274	0.440173	0.440665
	BET+ECFP	0.433186	0.444671	0.439946
	AE+BET+ECFP	0.430354	0.430661	0.436528

Continued on next page

Continued from previous page

Datasets	fingerprint	GBDT	SVM	RF
CHEMBL1741186	BET	0.85582	0.80547	0.944959
	AE	0.882751	0.800492	0.948399
	ECFP	0.836072	0.877429	0.968673
	AE+BET	0.857058	0.786611	0.935902
	AE+ECFP	0.819058	0.798077	0.935195
	BET+ECFP	0.813466	0.802525	0.937857
	AE+BET+ECFP	0.820423	0.784536	0.930327
CHEMBL1741200	BET	0.47605	0.488411	0.483663
	AE	0.466237	0.475793	0.475561
	ECFP	0.486901	0.516894	0.488365
	AE+BET	0.468541	0.478299	0.47767
	AE+ECFP	0.466018	0.482439	0.476334
	BET+ECFP	0.471595	0.488355	0.481531
	AE+BET+ECFP	0.466113	0.479343	0.477162
CHEMBL1741213	BET	0.49262	0.506441	0.493988
	AE	0.491891	0.507955	0.495574
	ECFP	0.506002	0.513404	0.492972
	AE+BET	0.488522	0.499164	0.492588
	AE+ECFP	0.485672	0.496664	0.489896
	BET+ECFP	0.485606	0.494993	0.489346
	AE+BET+ECFP	0.483127	0.492765	0.489418
CHEMBL2424504	BET	0.992797	0.954348	1.051876
	AE	1.013012	0.967264	1.046449
	ECFP	0.952967	1.005137	0.966886
	AE+BET	0.990399	0.946231	1.025614
	AE+ECFP	0.947273	0.950184	0.970621
	BET+ECFP	0.944457	0.939215	0.976581
	AE+BET+ECFP	0.952242	0.934798	0.980614
CHEMBL3392948	BET	0.940782	0.912443	0.993268
	AE	0.953817	0.916456	0.984409
	ECFP	0.969855	1.029836	0.952247
	AE+BET	0.939766	0.898739	0.980067
	AE+ECFP	0.9177	0.915545	0.933724
	BET+ECFP	0.913238	0.91483	0.941399
	AE+BET+ECFP	0.912986	0.894332	0.943516
CHEMBL3714079	BET	1.9075	1.892875	1.989625
	AE	1.988664	1.902061	2.00951
	ECFP	2.043532	2.170154	2.052835
	AE+BET	1.926683	1.862977	1.966032
	AE+ECFP	1.91657	1.915634	1.97455
	BET+ECFP	1.898664	1.907584	1.974468
	AE+BET+ECFP	1.891259	1.862092	1.95618

Table S5: Comparison of Pearson correlation coefficient (P) results among GBDT, SVM, and RF models

Datasets	fingerprint	GBDT	SVM	RF
CHEMBL216	BET	0.814999	0.839398	0.761530
	AE	0.800270	0.835006	0.753647
	ECFP	0.840992	0.811243	0.751852
	AE+BET	0.818351	0.845837	0.776665
	AE+ECFP	0.845689	0.844445	0.790532
	BET+ECFP	0.848435	0.847739	0.788076
	AE+BET+ECFP	0.844760	0.851772	0.795974
CHEMBL1801	BET	0.869587	0.893137	0.821903
	AE	0.835354	0.880908	0.746159
	ECFP	0.892900	0.854740	0.840037
	AE+BET	0.868431	0.894743	0.822192
	AE+ECFP	0.908016	0.886399	0.841610
	BET+ECFP	0.893337	0.891680	0.835505
	AE+BET+ECFP	0.896580	0.898870	0.840857
CHEMBL1978	BET	0.835505	0.840857	0.770439
	AE	0.816620	0.827680	0.748870
	ECFP	0.855630	0.816620	0.802780
	AE+BET	0.835505	0.843590	0.774620
	AE+ECFP	0.860880	0.845680	0.804560
	BET+ECFP	0.856560	0.845680	0.799240
	AE+BET+ECFP	0.836024	0.844433	0.784656
CHEMBL1856	BET	0.826284	0.840607	0.788254
	AE	0.813142	0.830531	0.794482
	ECFP	0.866043	0.829239	0.847758
	AE+BET	0.828664	0.843459	0.806381
	AE+ECFP	0.862876	0.851157	0.847333
	BET+ECFP	0.864985	0.852051	0.843968
	AE+BET+ECFP	0.858756	0.854012	0.840990
CHEMBL1995	BET	0.723485	0.712167	0.714004
	AE	0.745811	0.752306	0.715311
	ECFP	0.698636	0.642257	0.676503
	AE+BET	0.744565	0.745745	0.737460
	AE+ECFP	0.722790	0.719790	0.711640
	BET+ECFP	0.707830	0.676760	0.695810
	AE+BET+ECFP	0.722790	0.760660	0.715310
CHEMBL2047	BET	0.722792	0.717874	0.680469
	AE	0.675546	0.705493	0.657043
	ECFP	0.719746	0.69586	0.71969
	AE+BET	0.713044	0.722046	0.689619
	AE+ECFP	0.726548	0.726916	0.726378
	BET+ECFP	0.737328	0.730422	0.729984
	AE+BET+ECFP	0.733298	0.733242	0.727025

Continued on next page

Continued from previous page

Datasets	fingerprint	GBDT	SVM	RF
CHEMBL2068	BET	0.749483	0.777996	0.729095
	AE	0.71948	0.730602	0.662602
	ECFP	0.78496	0.768679	0.754575
	AE+BET	0.749674	0.774046	0.722961
	AE+ECFP	0.786863	0.780146	0.750255
	BET+ECFP	0.788628	0.793662	0.760193
	AE+BET+ECFP	0.783969	0.790687	0.754365
CHEMBL2073	BET	0.717901	0.784955	0.670063
	AE	0.734031	0.767338	0.679509
	ECFP	0.779804	0.735895	0.732112
	AE+BET	0.752738	0.78608	0.710813
	AE+ECFP	0.79692	0.774423	0.75402
	BET+ECFP	0.774419	0.77762	0.736929
	AE+BET+ECFP	0.786049	0.785152	0.749426
CHEMBL2083	BET	0.810449	0.824149	0.786657
	AE	0.790688	0.81775	0.760316
	ECFP	0.816993	0.79895	0.837375
	AE+BET	0.807717	0.827141	0.785209
	AE+ECFP	0.822682	0.825522	0.825708
	BET+ECFP	0.828418	0.830404	0.832936
	AE+BET+ECFP	0.824843	0.832109	0.822193
CHEMBL2085	BET	0.681515	0.65722	0.66796
	AE	0.647213	0.627021	0.597687
	ECFP	0.661867	0.643577	0.642478
	AE+BET	0.675533	0.652078	0.653871
	AE+ECFP	0.677247	0.659461	0.648374
	BET+ECFP	0.692293	0.675052	0.674095
	AE+BET+ECFP	0.689348	0.66842	0.667192
CHEMBL2107	BET	0.679853	0.696547	0.625978
	AE	0.656597	0.67614	0.619028
	ECFP	0.713039	0.665982	0.63987
	AE+BET	0.685684	0.702747	0.637446
	AE+ECFP	0.723978	0.707423	0.662663
	BET+ECFP	0.725145	0.716226	0.659574
	AE+BET+ECFP	0.72307	0.718558	0.663213
CHEMBL2147	BET	0.908495	0.924461	0.870382
	AE	0.902638	0.923936	0.801518
	ECFP	0.918862	0.8981	0.87741
	AE+BET	0.911844	0.928076	0.863142
	AE+ECFP	0.92429	0.924492	0.873603
	BET+ECFP	0.923994	0.924492	0.888307
	AE+BET+ECFP	0.92363	0.929	0.883788

Continued on next page

Continued from previous page

Datasets	fingerprint	GBDT	SVM	RF
CHEMBL2319	BET	0.694905	0.720465	0.701926
	AE	0.673872	0.735767	0.641352
	ECFP	0.716565	0.692502	0.671408
	AE+BET	0.696671	0.736108	0.693108
	AE+ECFP	0.719435	0.73385	0.678899
	BET+ECFP	0.722652	0.727574	0.708891
	AE+BET+ECFP	0.719637	0.737871	0.701625
CHEMBL2434	BET	0.786966	0.803634	0.760888
	AE	0.780729	0.808271	0.651859
	ECFP	0.790252	0.760366	0.754976
	AE+BET	0.794551	0.814208	0.746705
	AE+ECFP	0.806733	0.805851	0.755527
	BET+ECFP	0.803734	0.803864	0.77387
	AE+BET+ECFP	0.807508	0.813806	0.7708
CHEMBL2525	BET	0.794994	0.825899	0.760122
	AE	0.801328	0.817608	0.763123
	ECFP	0.820511	0.80782	0.752427
	AE+BET	0.806124	0.829597	0.773935
	AE+ECFP	0.829177	0.831377	0.779777
	BET+ECFP	0.825724	0.835518	0.778892
	AE+BET+ECFP	0.826089	0.837234	0.78459
CHEMBL2593	BET	0.764038	0.782056	0.739553
	AE	0.760605	0.821301	0.736061
	ECFP	0.801727	0.757598	0.765037
	AE+BET	0.774255	0.811698	0.754591
	AE+ECFP	0.809296	0.814689	0.777671
	BET+ECFP	0.806989	0.799874	0.772769
	AE+BET+ECFP	0.805096	0.816184	0.776137
CHEMBL2717	BET	0.733945	0.747649	0.724335
	AE	0.734027	0.749707	0.662407
	ECFP	0.75555	0.740043	0.744095
	AE+BET	0.742582	0.754752	0.717736
	AE+ECFP	0.758574	0.756777	0.730405
	BET+ECFP	0.757594	0.756155	0.750075
	AE+BET+ECFP	0.757216	0.759419	0.740215
CHEMBL2730	BET	0.74805	0.761896	0.708827
	AE	0.747058	0.77321	0.72855
	ECFP	0.740482	0.689374	0.746266
	AE+BET	0.756544	0.776851	0.733427
	AE+ECFP	0.760221	0.761159	0.754164
	BET+ECFP	0.763679	0.757117	0.746889
	AE+BET+ECFP	0.766249	0.773498	0.750903

Continued on next page

Continued from previous page

Datasets	fingerprint	GBDT	SVM	RF
CHEMBL2778	BET	0.828193	0.836262	0.805531
	AE	0.821868	0.833645	0.772369
	ECFP	0.786216	0.770859	0.780476
	AE+BET	0.832535	0.845154	0.79976
	AE+ECFP	0.818094	0.828774	0.794348
	BET+ECFP	0.819999	0.829629	0.805423
	AE+BET+ECFP	0.827955	0.841997	0.804812
CHEMBL2789	BET	0.922433	0.923565	0.899934
	AE	0.921733	0.924342	0.90022
	ECFP	0.935849	0.916527	0.919621
	AE+BET	0.925487	0.927451	0.911068
	AE+ECFP	0.936594	0.930917	0.922068
	BET+ECFP	0.936501	0.930639	0.918765
	AE+BET+ECFP	0.935088	0.932473	0.920904
CHEMBL2889	BET	0.686965	0.709429	0.660867
	AE	0.639482	0.699387	0.609046
	ECFP	0.690019	0.643043	0.700391
	AE+BET	0.676303	0.715765	0.648357
	AE+ECFP	0.695834	0.692644	0.689077
	BET+ECFP	0.707366	0.698263	0.702576
	AE+BET+ECFP	0.701768	0.708678	0.689303
CHEMBL2959	BET	0.75895	0.775073	0.713241
	AE	0.753654	0.776577	0.672758
	ECFP	0.791121	0.764369	0.703616
	AE+BET	0.768159	0.786136	0.719622
	AE+ECFP	0.797522	0.796104	0.725187
	BET+ECFP	0.797835	0.794037	0.733094
	AE+BET+ECFP	0.795624	0.798564	0.736439
CHEMBL3018	BET	0.778921	0.815835	0.746140
	AE	0.730191	0.792276	0.662761
	ECFP	0.781483	0.767339	0.735008
	AE+BET	0.772000	0.815759	0.732673
	AE+ECFP	0.789835	0.802518	0.733108
	BET+ECFP	0.804776	0.815711	0.762041
	AE+BET+ECFP	0.798625	0.818042	0.752353
CHEMBL3060	BET	0.707287	0.736221	0.664890
	AE	0.715411	0.729822	0.676391
	ECFP	0.674647	0.692368	0.731650
	AE+BET	0.722291	0.743000	0.689628
	AE+ECFP	0.724187	0.737618	0.738781
	BET+ECFP	0.713168	0.741296	0.726709
	AE+BET+ECFP	0.729070	0.747367	0.729219

Continued on next page

Continued from previous page

Datasets	fingerprint	GBDT	SVM	RF
CHEMBL3100	BET	0.754257	0.750823	0.727558
	AE	0.732505	0.764612	0.650419
	ECFP	0.782122	0.711233	0.746928
	AE+BET	0.751506	0.764558	0.721424
	AE+ECFP	0.783338	0.767404	0.744064
	BET+ECFP	0.789939	0.762076	0.759806
	AE+BET+ECFP	0.781842	0.771703	0.753065
CHEMBL3106	BET	0.674253	0.742252	0.641318
	AE	0.663186	0.761929	0.562033
	ECFP	0.592488	0.613324	0.633270
	AE+BET	0.682371	0.759659	0.632269
	AE+ECFP	0.648839	0.725411	0.627508
	BET+ECFP	0.660920	0.715850	0.675663
	AE+BET+ECFP	0.672812	0.742237	0.657666
CHEMBL3119	BET	0.804448	0.832304	0.788992
	AE	0.806024	0.847535	0.774778
	ECFP	0.840993	0.807787	0.794584
	AE+BET	0.812434	0.847093	0.795056
	AE+ECFP	0.842389	0.847125	0.803363
	BET+ECFP	0.837484	0.841048	0.805066
	AE+BET+ECFP	0.835883	0.850589	0.806479
CHEMBL3194	BET	0.616154	0.627781	0.599965
	AE	0.610900	0.568198	0.559975
	ECFP	0.580254	0.550039	0.589507
	AE+BET	0.628940	0.611833	0.604265
	AE+ECFP	0.617126	0.581785	0.606379
	BET+ECFP	0.615294	0.609802	0.624272
	AE+BET+ECFP	0.627626	0.607968	0.622809
CHEMBL3286	BET	0.785126	0.804883	0.733077
	AE	0.760763	0.801652	0.704127
	ECFP	0.821946	0.782852	0.730148
	AE+BET	0.786669	0.813111	0.739736
	AE+ECFP	0.823717	0.819270	0.754985
	BET+ECFP	0.829246	0.822158	0.760601
	AE+BET+ECFP	0.822901	0.825226	0.763018
CHEMBL3359	BET	0.773233	0.795452	0.745955
	AE	0.757570	0.795635	0.733186
	ECFP	0.820213	0.747459	0.805358
	AE+BET	0.778687	0.805129	0.754789
	AE+ECFP	0.818516	0.797548	0.804196
	BET+ECFP	0.817642	0.794929	0.801735
	AE+BET+ECFP	0.813008	0.805771	0.796518

Continued on next page

Continued from previous page

Datasets	fingerprint	GBDT	SVM	RF
CHEMBL3401	BET	0.764036	0.793471	0.724633
	AE	0.749451	0.783539	0.718135
	ECFP	0.693911	0.657705	0.662625
	AE+BET	0.767901	0.795674	0.739238
	AE+ECFP	0.753880	0.770290	0.722356
	BET+ECFP	0.759988	0.778141	0.723664
	AE+BET+ECFP	0.770960	0.791391	0.739399
CHEMBL3475	BET	0.690040	0.726352	0.670595
	AE	0.725778	0.747769	0.676139
	ECFP	0.661860	0.671501	0.669787
	AE+BET	0.722758	0.749974	0.693917
	AE+ECFP	0.714061	0.740005	0.704052
	BET+ECFP	0.697118	0.735825	0.690934
	AE+BET+ECFP	0.719035	0.751686	0.705269
CHEMBL3572	BET	0.817036	0.830816	0.797547
	AE	0.806900	0.831162	0.775394
	ECFP	0.818220	0.800087	0.801035
	AE+BET	0.821556	0.836506	0.800508
	AE+ECFP	0.832344	0.834856	0.809173
	BET+ECFP	0.833552	0.834467	0.812983
	AE+BET+ECFP	0.834874	0.839957	0.813271
CHEMBL3650	BET	0.850347	0.869155	0.800914
	AE	0.843298	0.870527	0.805710
	ECFP	0.875224	0.854477	0.773823
	AE+BET	0.855754	0.876402	0.817778
	AE+ECFP	0.880069	0.878949	0.815664
	BET+ECFP	0.879496	0.878261	0.810350
	AE+BET+ECFP	0.877555	0.882186	0.822309
CHEMBL3785	BET	0.792492	0.818953	0.766109
	AE	0.790694	0.814295	0.720349
	ECFP	0.817185	0.748611	0.785953
	AE+BET	0.798020	0.822527	0.775443
	AE+ECFP	0.821818	0.810949	0.795498
	BET+ECFP	0.820734	0.812970	0.798982
	AE+BET+ECFP	0.818716	0.822155	0.800998
CHEMBL3869	BET	0.730963	0.755053	0.691228
	AE	0.706666	0.766628	0.655374
	ECFP	0.784962	0.729314	0.731689
	AE+BET	0.734948	0.774933	0.691884
	AE+ECFP	0.785665	0.780220	0.729213
	BET+ECFP	0.789827	0.773429	0.737845
	AE+BET+ECFP	0.782303	0.784579	0.729969

Continued on next page

Continued from previous page

Datasets	fingerprint	GBDT	SVM	RF
CHEMBL4029	BET	0.710498	0.693105	0.683215
	AE	0.691647	0.716187	0.68052
	ECFP	0.677539	0.673857	0.697444
	AE+BET	0.710106	0.711485	0.692754
	AE+ECFP	0.712308	0.72319	0.715001
	BET+ECFP	0.717615	0.712071	0.716152
	AE+BET+ECFP	0.722514	0.72271	0.716206
CHEMBL4073	BET	0.81858	0.830704	0.789021
	AE	0.800763	0.82386	0.749561
	ECFP	0.81792	0.814467	0.816159
	AE+BET	0.818449	0.834296	0.786661
	AE+ECFP	0.827341	0.839101	0.812782
	BET+ECFP	0.834745	0.841523	0.826064
	AE+BET+ECFP	0.83254	0.843289	0.817373
CHEMBL4076	BET	0.533708	0.568782	0.473284
	AE	0.534977	0.534438	0.488671
	ECFP	0.514429	0.515644	0.542322
	AE+BET	0.551148	0.564919	0.509677
	AE+ECFP	0.572067	0.571384	0.603329
	BET+ECFP	0.567585	0.588777	0.588833
	AE+BET+ECFP	0.580815	0.586938	0.597679
CHEMBL4223	BET	0.470342	0.433258	0.454341
	AE	0.459136	0.448983	0.43957
	ECFP	0.540385	0.535281	0.518159
	AE+BET	0.482619	0.452656	0.467067
	AE+ECFP	0.542511	0.515091	0.518617
	BET+ECFP	0.539101	0.511425	0.520331
	AE+BET+ECFP	0.534723	0.500002	0.51455
CHEMBL4306	BET	0.78893	0.781003	0.772259
	AE	0.795801	0.777182	0.772417
	ECFP	0.795624	0.780959	0.780332
	AE+BET	0.799238	0.788142	0.782862
	AE+ECFP	0.812045	0.801408	0.79068
	BET+ECFP	0.808491	0.803896	0.791996
	AE+BET+ECFP	0.811628	0.803832	0.793298
CHEMBL4394	BET	0.762413	0.769413	0.749168
	AE	0.76798	0.765731	0.74953
	ECFP	0.771747	0.741093	0.773044
	AE+BET	0.770925	0.773548	0.756523
	AE+ECFP	0.78463	0.77249	0.776295
	BET+ECFP	0.780384	0.772004	0.775183
	AE+BET+ECFP	0.783015	0.777145	0.773773

Continued on next page

Continued from previous page

Datasets	fingerprint	GBDT	SVM	RF
CHEMBL4409	BET	0.796404	0.823931	0.718948
	AE	0.793357	0.831615	0.690588
	ECFP	0.826222	0.79202	0.63716
	AE+BET	0.806007	0.836294	0.725264
	AE+ECFP	0.837421	0.838244	0.714987
	BET+ECFP	0.834491	0.834184	0.712941
	AE+BET+ECFP	0.833911	0.842898	0.732013
CHEMBL4427	BET	0.849075	0.870118	0.823925
	AE	0.844259	0.867832	0.825593
	ECFP	0.863322	0.817061	0.854718
	AE+BET	0.853257	0.875987	0.837935
	AE+ECFP	0.869187	0.862326	0.858053
	BET+ECFP	0.872476	0.862411	0.859513
	AE+BET+ECFP	0.869958	0.872094	0.858072
CHEMBL4462	BET	0.847983	0.866955	0.76405
	AE	0.847825	0.870482	0.762267
	ECFP	0.877117	0.838782	0.82078
	AE+BET	0.858216	0.874756	0.802803
	AE+ECFP	0.883514	0.870952	0.836124
	BET+ECFP	0.879618	0.869931	0.822388
	AE+BET+ECFP	0.88001	0.876185	0.834054
CHEMBL4507	BET	0.167509	0.30744	0.18514
	AE	0.089148	0.085852	0.139251
	ECFP	0.338779	0.312432	0.179611
	AE+BET	0.133627	0.194063	0.170736
	AE+ECFP	0.276901	0.227199	0.171488
	BET+ECFP	0.314382	0.331158	0.197463
	AE+BET+ECFP	0.26464	0.257686	0.184596
CHEMBL4607	BET	0.741412	0.751027	0.718155
	AE	0.719601	0.758992	0.673698
	ECFP	0.736102	0.712206	0.732663
	AE+BET	0.745559	0.768277	0.721188
	AE+ECFP	0.759004	0.763101	0.737584
	BET+ECFP	0.758573	0.754377	0.743217
	AE+BET+ECFP	0.762928	0.769275	0.742687
CHEMBL4616	BET	0.713017	0.72117	0.671994
	AE	0.705683	0.719193	0.618299
	ECFP	0.719547	0.701481	0.636434
	AE+BET	0.724269	0.733321	0.667877
	AE+ECFP	0.741053	0.738684	0.657319
	BET+ECFP	0.741054	0.738201	0.677212
	AE+BET+ECFP	0.744568	0.744483	0.676499

Continued on next page

Continued from previous page

Datasets	fingerprint	GBDT	SVM	RF
CHEMBL4691	BET	0.763721	0.758828	0.745976
	AE	0.742744	0.765624	0.735638
	ECFP	0.769257	0.743443	0.767845
	AE+BET	0.760954	0.770407	0.748062
	AE+ECFP	0.781246	0.780687	0.77188
	BET+ECFP	0.788282	0.778116	0.776742
	AE+BET+ECFP	0.784275	0.783353	0.771584
CHEMBL4767	BET	0.69671	0.724522	0.653421
	AE	0.708148	0.741164	0.697077
	ECFP	0.688758	0.664707	0.694848
	AE+BET	0.716238	0.744717	0.696527
	AE+ECFP	0.7425	0.743638	0.744045
	BET+ECFP	0.733636	0.733153	0.717516
	AE+BET+ECFP	0.745608	0.750747	0.734516
CHEMBL4789	BET	0.769417	0.777399	0.737563
	AE	0.739287	0.764525	0.696693
	ECFP	0.719887	0.682526	0.692315
	AE+BET	0.7672	0.777778	0.73659
	AE+ECFP	0.757324	0.75945	0.727369
	BET+ECFP	0.768544	0.765199	0.740325
	AE+BET+ECFP	0.773448	0.775184	0.744549
CHEMBL4835	BET	0.824556	0.834419	0.790131
	AE	0.792757	0.837213	0.764187
	ECFP	0.842048	0.796234	0.798088
	AE+BET	0.821068	0.843147	0.797775
	AE+ECFP	0.842392	0.840695	0.815445
	BET+ECFP	0.848836	0.843263	0.818372
	AE+BET+ECFP	0.843912	0.848499	0.820353
CHEMBL5247	BET	0.518204	0.597614	0.487376
	AE	0.537235	0.618817	0.484693
	ECFP	0.502921	0.523037	0.476656
	AE+BET	0.543946	0.619221	0.507756
	AE+ECFP	0.550316	0.612044	0.520315
	BET+ECFP	0.541532	0.600688	0.523103
	AE+BET+ECFP	0.555858	0.619425	0.529686
CHEMBL5314	BET	0.808889	0.816315	0.778122
	AE	0.778812	0.827038	0.685639
	ECFP	0.803641	0.753489	0.747473
	AE+BET	0.807863	0.830645	0.764412
	AE+ECFP	0.824639	0.816826	0.765533
	BET+ECFP	0.827359	0.81093	0.787075
	AE+BET+ECFP	0.828547	0.826434	0.78539

Continued on next page

Continued from previous page

Datasets	fingerprint	GBDT	SVM	RF
CHEMBL5319	BET	0.58673	0.586488	0.570538
	AE	0.611181	0.604662	0.606212
	ECFP	0.629802	0.625717	0.648679
	AE+BET	0.606598	0.604263	0.599422
	AE+ECFP	0.642517	0.635674	0.655001
	BET+ECFP	0.632965	0.628982	0.644789
	AE+BET+ECFP	0.63493	0.628744	0.642894
CHEMBL5378	BET	0.539492	0.569901	0.521768
	AE	0.435036	0.540081	0.468002
	ECFP	0.601721	0.555178	0.593924
	AE+BET	0.512318	0.568484	0.512395
	AE+ECFP	0.584985	0.579755	0.577713
	BET+ECFP	0.604152	0.593272	0.588295
	AE+BET+ECFP	0.584416	0.588914	0.571461
CHEMBL5398	BET	-0.343812	-0.093295	-0.361124
	AE	0.000539	0.114932	0.177764
	ECFP	0.292921	0.1973	0.268895
	AE+BET	-0.158604	0.016418	-0.047822
	AE+ECFP	0.194157	0.164511	0.260963
	BET+ECFP	0.075291	0.055831	-0.020506
	AE+BET+ECFP	0.051562	0.081278	0.083604
CHEMBL5480	BET	0.564164	0.545113	0.540535
	AE	0.595957	0.556133	0.570505
	ECFP	0.56153	0.554731	0.570093
	AE+BET	0.590354	0.563456	0.564772
	AE+ECFP	0.600954	0.591468	0.588077
	BET+ECFP	0.590867	0.58916	0.577805
	AE+BET+ECFP	0.602573	0.590536	0.583542
CHEMBL5493	BET	0.783784	0.77563	0.76516
	AE	0.792734	0.800657	0.769695
	ECFP	0.799545	0.752562	0.773868
	AE+BET	0.794005	0.796481	0.779077
	AE+ECFP	0.817441	0.801421	0.794391
	BET+ECFP	0.810401	0.786431	0.784979
	AE+BET+ECFP	0.812858	0.800864	0.792162
CHEMBL5918	BET	-0.008035	0.026553	-0.08686
	AE	0.08459	0.018198	0.200001
	ECFP	-0.028696	0.002037	0.147333
	AE+BET	0.045208	0.023613	0.074324
	AE+ECFP	0.046502	0.013626	0.207318
	BET+ECFP	-0.018923	0.018902	0.037664
	AE+BET+ECFP	0.028106	0.019669	0.114524

Continued on next page

Continued from previous page

Datasets	fingerprint	GBDT	SVM	RF
CHEMBL6003	BET	0.381774	0.378387	0.226039
	AE	0.284758	0.333347	0.198774
	ECFP	0.384013	0.441862	0.265659
	AE+BET	0.363751	0.373249	0.24939
	AE+ECFP	0.370598	0.426468	0.281591
	BET+ECFP	0.420521	0.435408	0.297353
	AE+BET+ECFP	0.400213	0.419865	0.29906
CHEMBL6007	BET	0.77021	0.763632	0.760147
	AE	0.736553	0.748283	0.721714
	ECFP	0.752639	0.743374	0.7331
	AE+BET	0.765766	0.7616	0.757349
	AE+ECFP	0.77014	0.767121	0.752741
	BET+ECFP	0.777253	0.773979	0.762101
	AE+BET+ECFP	0.77756	0.772608	0.764032
CHEMBL1250348	BET	0.79131	0.847619	0.654302
	AE	0.809289	0.850953	0.600159
	ECFP	0.837072	0.792928	0.843582
	AE+BET	0.811963	0.856446	0.678448
	AE+ECFP	0.845878	0.84737	0.798587
	BET+ECFP	0.834606	0.843398	0.807796
	AE+BET+ECFP	0.837931	0.855057	0.786078
CHEMBL1293255	BET	0.788229	0.800844	0.765582
	AE	0.784874	0.797569	0.754157
	ECFP	0.777404	0.750688	0.757013
	AE+BET	0.795316	0.806396	0.772288
	AE+ECFP	0.799511	0.795418	0.7764
	BET+ECFP	0.798822	0.797098	0.776013
	AE+BET+ECFP	0.803242	0.805447	0.780756
CHEMBL1293293	BET	0.742614	0.778622	0.715673
	AE	0.753746	0.775552	0.710672
	ECFP	0.769798	0.70063	0.760187
	AE+BET	0.763517	0.790047	0.731943
	AE+ECFP	0.791076	0.776612	0.768352
	BET+ECFP	0.780933	0.776626	0.7678
	AE+BET+ECFP	0.787869	0.790321	0.766838
CHEMBL1741179	BET	0.580596	0.583908	0.573592
	AE	0.566185	0.604684	0.562439
	ECFP	0.526505	0.471866	0.518755
	AE+BET	0.587359	0.609051	0.582748
	AE+ECFP	0.572303	0.579972	0.565685
	BET+ECFP	0.579256	0.568361	0.568318
	AE+BET+ECFP	0.588397	0.595309	0.579622

Continued on next page

Continued from previous page

Datasets	fingerprint	GBDT	SVM	RF
CHEMBL1741186	BET	0.728446	0.759776	0.678189
	AE	0.715542	0.762949	0.665456
	ECFP	0.739401	0.707806	0.637567
	AE+BET	0.73492	0.772199	0.691478
	AE+ECFP	0.757369	0.767891	0.693507
	BET+ECFP	0.757983	0.764825	0.692545
	AE+BET+ECFP	0.758829	0.776226	0.704503
CHEMBL1741200	BET	0.327942	0.309365	0.279288
	AE	0.377265	0.353147	0.32861
	ECFP	0.328876	0.278131	0.258785
	AE+BET	0.365913	0.341251	0.316592
	AE+ECFP	0.380208	0.342933	0.324759
	BET+ECFP	0.355621	0.317497	0.29188
	AE+BET+ECFP	0.377966	0.340496	0.320163
CHEMBL1741213	BET	0.345277	0.341889	0.342159
	AE	0.352387	0.341105	0.331195
	ECFP	0.360833	0.329373	0.342931
	AE+BET	0.365221	0.358207	0.352818
	AE+ECFP	0.385966	0.363397	0.366594
	BET+ECFP	0.384598	0.366746	0.371969
	AE+BET+ECFP	0.390724	0.372207	0.373762
CHEMBL2424504	BET	0.76953	0.783853	0.734688
	AE	0.767414	0.777242	0.736228
	ECFP	0.785845	0.765812	0.777684
	AE+BET	0.777161	0.788136	0.753249
	AE+ECFP	0.792258	0.789975	0.777631
	BET+ECFP	0.791533	0.795121	0.775205
	AE+BET+ECFP	0.791703	0.796122	0.775492
CHEMBL3392948	BET	0.683831	0.708852	0.637271
	AE	0.678935	0.705665	0.651879
	ECFP	0.678936	0.663321	0.6746
	AE+BET	0.688687	0.717348	0.654527
	AE+ECFP	0.70196	0.714552	0.692211
	BET+ECFP	0.706235	0.716722	0.68354
	AE+BET+ECFP	0.706894	0.724348	0.685668
CHEMBL3714079	BET	0.724035	0.731569	0.692873
	AE	0.699587	0.728769	0.685114
	ECFP	0.680925	0.652365	0.666786
	AE+BET	0.721476	0.739684	0.704107
	AE+ECFP	0.71963	0.725505	0.699209
	BET+ECFP	0.724871	0.72857	0.698889
	AE+BET+ECFP	0.730449	0.740695	0.707632

Table S6: Comparison of coefficient of determination R^2 results among GBDT, SVM, and RF models

Datasets	fingerprint	GBDT	SVM	RF
CHEMBL216	BET	0.651119	0.704554	0.550389
	AE	0.614196	0.697198	0.535729
	ECFP	0.706688	0.657334	0.552034
	AE_BET	0.644919	0.715314	0.55967
	AE_ECFP	0.702264	0.71049	0.579189
	BET_ECFP	0.712889	0.715854	0.581327
	AE_BET_ECFP	0.696476	0.723061	0.58251
CHEMBL1801	BET	0.737214	0.797255	0.652892
	AE	0.64158	0.77588	0.519662
	ECFP	0.79724	0.728113	0.705241
	AE_BET	0.708263	0.799645	0.6207
	AE_ECFP	0.767276	0.783169	0.673346
	BET_ECFP	0.789905	0.791882	0.703147
	AE_BET_ECFP	0.765082	0.799727	0.678998
CHEMBL1836	BET	0.632931	0.695396	0.533464
	AE	0.614367	0.683263	0.548149
	ECFP	0.675287	0.621558	0.531358
	AE_BET	0.632957	0.70179	0.556092
	AE_ECFP	0.682465	0.689256	0.569934
	BET_ECFP	0.685117	0.693847	0.560848
	AE_BET_ECFP	0.675508	0.704373	0.570503
CHEMBL1856	BET	0.663353	0.706516	0.602956
	AE	0.639493	0.689691	0.617318
	ECFP	0.747161	0.642323	0.716597
	AE_BET	0.661258	0.710949	0.626582
	AE_ECFP	0.730752	0.708876	0.704377
	BET_ECFP	0.736844	0.711256	0.697212
	AE_BET_ECFP	0.718379	0.720644	0.686202
CHEMBL1995	BET	0.498783	0.519675	0.482056
	AE	0.513488	0.565705	0.457109
	ECFP	0.4878	0.377705	0.455477
	AE_BET	0.515156	0.555765	0.487122
	AE_ECFP	0.531505	0.521349	0.486776
	BET_ECFP	0.522894	0.498012	0.49564
	AE_BET_ECFP	0.530905	0.537491	0.498167
CHEMBL2047	BET	0.506139	0.512549	0.447305
	AE	0.430192	0.492167	0.416436
	ECFP	0.510251	0.482023	0.517016
	AE_BET	0.48082	0.519586	0.449776
	AE_ECFP	0.520356	0.528281	0.514974
	BET_ECFP	0.541009	0.533297	0.522602
	AE_BET_ECFP	0.524684	0.537546	0.507628

Continued on next page

Continued from previous page

Datasets	fingerprint	GBDT	SVM	RF
CHEMBL2068	BET	0.553196	0.604547	0.518715
	AE	0.486828	0.533765	0.422209
	ECFP	0.615493	0.587383	0.56855
	AE_BET	0.537025	0.597425	0.4956
	AE_ECFP	0.601278	0.601107	0.540921
	BET_ECFP	0.616215	0.625814	0.56716
	AE_BET_ECFP	0.59584	0.619076	0.545027
CHEMBL2073	BET	0.48343	0.591547	0.423071
	AE	0.498653	0.578452	0.444934
	ECFP	0.60411	0.479519	0.533886
	AE_BET	0.511715	0.596194	0.463006
	AE_ECFP	0.597326	0.566596	0.546651
	BET_ECFP	0.57416	0.564655	0.522266
	AE_BET_ECFP	0.571845	0.584473	0.525089
CHEMBL2083	BET	0.639124	0.678992	0.604689
	AE	0.592531	0.668019	0.540594
	ECFP	0.666725	0.630579	0.700723
	AE_BET	0.623767	0.683251	0.586329
	AE_ECFP	0.660591	0.673752	0.659623
	BET_ECFP	0.676823	0.682604	0.682791
	AE_BET_ECFP	0.660705	0.68676	0.652107
CHEMBL2085	BET	0.440048	0.428465	0.416365
	AE	0.391717	0.387716	0.340055
	ECFP	0.434515	0.413189	0.412288
	AE_BET	0.424855	0.423099	0.392573
	AE_ECFP	0.449457	0.434089	0.407391
	BET_ECFP	0.471192	0.454551	0.439561
	AE_BET_ECFP	0.457304	0.446398	0.421043
CHEMBL2107	BET	0.454351	0.482083	0.364323
	AE	0.40282	0.453994	0.336508
	ECFP	0.500728	0.437232	0.403926
	AE_BET	0.445351	0.493238	0.360125
	AE_ECFP	0.509647	0.495821	0.399346
	BET_ECFP	0.520095	0.508897	0.407392
	AE_BET_ECFP	0.504719	0.513168	0.395855
CHEMBL2147	BET	0.817985	0.854164	0.741969
	AE	0.794643	0.853291	0.637632
	ECFP	0.844029	0.803068	0.766785
	AE_BET	0.816313	0.860718	0.723425
	AE_ECFP	0.844759	0.851398	0.74768
	BET_ECFP	0.849233	0.851375	0.775418
	AE_BET_ECFP	0.842729	0.860382	0.759967

Continued on next page

Continued from previous page

Datasets	fingerprint	GBDT	SVM	RF
CHEMBL2319	BET	0.471215	0.5188	0.468063
	AE	0.429312	0.539993	0.39985
	ECFP	0.51039	0.47394	0.447742
	AE_BET	0.462934	0.54027	0.45202
	AE_ECFP	0.507571	0.531576	0.45351
	BET_ECFP	0.516249	0.524208	0.488924
	AE_BET_ECFP	0.504011	0.539053	0.473573
CHEMBL2434	BET	0.612021	0.644659	0.564361
	AE	0.585709	0.652628	0.411644
	ECFP	0.620969	0.540131	0.569286
	AE_BET	0.61229	0.662755	0.527526
	AE_ECFP	0.64217	0.640367	0.548934
	BET_ECFP	0.642958	0.635341	0.58814
	AE_BET_ECFP	0.641219	0.657382	0.568123
CHEMBL2525	BET	0.625233	0.681914	0.568954
	AE	0.614761	0.668278	0.553296
	ECFP	0.671851	0.648651	0.564552
	AE_BET	0.630896	0.688057	0.575083
	AE_ECFP	0.678066	0.688823	0.586839
	BET_ECFP	0.678629	0.695459	0.5947
	AE_BET_ECFP	0.670947	0.698946	0.593299
CHEMBL2593	BET	0.575338	0.61007	0.534052
	AE	0.557134	0.674012	0.535636
	ECFP	0.641546	0.545705	0.581427
	AE_BET	0.580252	0.658698	0.55352
	AE_ECFP	0.642772	0.654635	0.591642
	BET_ECFP	0.644343	0.631912	0.58239
	AE_BET_ECFP	0.632828	0.661244	0.584344
CHEMBL2717	BET	0.531503	0.558895	0.519834
	AE	0.518051	0.56145	0.427418
	ECFP	0.569291	0.54577	0.550084
	AE_BET	0.534523	0.569344	0.498176
	AE_ECFP	0.566697	0.572524	0.522077
	BET_ECFP	0.569779	0.571755	0.557493
	AE_BET_ECFP	0.562795	0.576486	0.53485
CHEMBL2730	BET	0.548846	0.579251	0.49311
	AE	0.532591	0.597209	0.51455
	ECFP	0.541236	0.452363	0.556456
	AE_BET	0.550479	0.60328	0.518601
	AE_ECFP	0.571636	0.576013	0.557887
	BET_ECFP	0.579644	0.569801	0.549131
	AE_BET_ECFP	0.576041	0.596395	0.548707

Continued on next page

Continued from previous page

Datasets	fingerprint	GBDT	SVM	RF
CHEMBL2778	BET	0.674124	0.698984	0.638437
	AE	0.656411	0.694802	0.586461
	ECFP	0.611773	0.546826	0.606388
	AE_BET	0.674356	0.713535	0.625091
	AE_ECFP	0.664435	0.675997	0.625744
	BET_ECFP	0.669677	0.675449	0.64498
	AE_BET_ECFP	0.67684	0.702146	0.639108
CHEMBL2789	BET	0.843367	0.852693	0.805753
	AE	0.836467	0.853877	0.799428
	ECFP	0.875486	0.830066	0.845464
	AE_BET	0.845072	0.85945	0.819011
	AE_ECFP	0.871616	0.863146	0.843411
	BET_ECFP	0.873421	0.862779	0.840899
	AE_BET_ECFP	0.867235	0.867207	0.840293
CHEMBL2889	BET	0.461043	0.501532	0.423965
	AE	0.391141	0.486829	0.359054
	ECFP	0.461449	0.401801	0.487917
	AE_BET	0.437775	0.511485	0.40238
	AE_ECFP	0.480518	0.477528	0.467258
	BET_ECFP	0.499294	0.485457	0.488457
	AE_BET_ECFP	0.484079	0.500858	0.462382
CHEMBL2959	BET	0.564166	0.599595	0.48528
	AE	0.538165	0.60197	0.393151
	ECFP	0.624353	0.577384	0.487352
	AE_BET	0.563966	0.61778	0.46031
	AE_ECFP	0.620273	0.628916	0.474743
	BET_ECFP	0.627749	0.626103	0.510232
	AE_BET_ECFP	0.613474	0.634694	0.490595
CHEMBL3018	BET	0.594113	0.665564	0.546051
	AE	0.51191	0.627177	0.409613
	ECFP	0.600914	0.541342	0.539382
	AE+BET	0.570605	0.66522	0.507956
	AE+ECFP	0.608614	0.632537	0.51592
	BET+ECFP	0.638457	0.652341	0.570113
	AE+BET+ECFP	0.618196	0.662923	0.542324
CHEMBL3060	BET	0.472446	0.541688	0.412521
	AE	0.474317	0.531467	0.413785
	ECFP	0.45086	0.446159	0.530927
	AE+BET	0.482074	0.550108	0.426352
	AE+ECFP	0.50895	0.527177	0.512189
	BET+ECFP	0.49918	0.532577	0.505244
	AE+BET+ECFP	0.507021	0.546682	0.490879

Continued on next page

Continued from previous page

Datasets	fingerprint	GBDT	SVM	RF
CHEMBL3100	BET	0.560188	0.563654	0.522594
	AE	0.515347	0.584238	0.3835
	ECFP	0.597805	0.420774	0.551545
	AE+BET	0.54744	0.584177	0.490543
	AE+ECFP	0.5933	0.559975	0.523596
	BET+ECFP	0.608367	0.553825	0.564032
	AE+BET+ECFP	0.591455	0.58036	0.539449
CHEMBL3106	BET	0.4333	0.54671	0.402437
	AE	0.391055	0.57491	0.301707
	ECFP	0.344335	0.369947	0.388813
	AE+BET	0.423212	0.570601	0.37511
	AE+ECFP	0.399425	0.509865	0.371878
	BET+ECFP	0.42106	0.497492	0.43334
	AE+BET+ECFP	0.4229	0.535585	0.40315
CHEMBL3119	BET	0.63918	0.692266	0.616267
	AE	0.634773	0.717949	0.597033
	ECFP	0.706879	0.625285	0.631209
	AE+BET	0.645878	0.716992	0.623483
	AE+ECFP	0.701962	0.709023	0.641915
	BET+ECFP	0.697306	0.698608	0.644558
	AE+BET+ECFP	0.688861	0.71811	0.643924
CHEMBL3194	BET	0.367303	0.393054	0.347716
	AE	0.353014	0.322161	0.301969
	ECFP	0.325206	0.269246	0.341924
	AE+BET	0.372583	0.373389	0.34237
	AE+ECFP	0.37709	0.329654	0.359922
	BET+ECFP	0.376441	0.36174	0.38101
	AE+BET+ECFP	0.384326	0.363852	0.371289
CHEMBL3286	BET	0.590535	0.64662	0.487751
	AE	0.538847	0.642088	0.441144
	ECFP	0.674429	0.611693	0.518349
	AE+BET	0.577314	0.660671	0.47827
	AE+ECFP	0.656057	0.667048	0.512081
	BET+ECFP	0.672681	0.672459	0.528999
	AE+BET+ECFP	0.646711	0.677812	0.514462
CHEMBL3359	BET	0.582916	0.632343	0.540802
	AE	0.556166	0.632987	0.524434
	ECFP	0.672392	0.521039	0.646867
	AE+BET	0.584494	0.647209	0.548488
	AE+ECFP	0.655707	0.622935	0.627937
	BET+ECFP	0.658344	0.617958	0.626255
	AE+BET+ECFP	0.642523	0.64067	0.610958

Continued on next page

Continued from previous page

Datasets	fingerprint	GBDT	SVM	RF
CHEMBL3401	BET	0.55658	0.629373	0.505658
	AE	0.531108	0.613841	0.48069
	ECFP	0.480876	0.360501	0.436103
	AE+BET	0.554258	0.632572	0.508812
	AE+ECFP	0.548617	0.561257	0.504309
	BET+ECFP	0.56006	0.571299	0.512374
	AE+BET+ECFP	0.564797	0.606311	0.519948
CHEMBL3475	BET	0.455484	0.527573	0.429218
	AE	0.497041	0.556724	0.442739
	ECFP	0.428712	0.446454	0.445355
	AE+BET	0.488627	0.560231	0.453277
	AE+ECFP	0.5027	0.544328	0.475883
	BET+ECFP	0.48125	0.538991	0.459559
	AE+BET+ECFP	0.501008	0.560383	0.47084
CHEMBL3572	BET	0.662876	0.688387	0.623
	AE	0.604492	0.689899	0.583693
	ECFP	0.665407	0.630523	0.640972
	AE+BET	0.651259	0.698898	0.619243
	AE+ECFP	0.680994	0.69273	0.642155
	BET+ECFP	0.692972	0.691747	0.652149
	AE+BET+ECFP	0.685348	0.702743	0.645169
CHEMBL3650	BET	0.710599	0.755076	0.609711
	AE	0.683708	0.757593	0.629341
	ECFP	0.765546	0.727942	0.592212
	AE+BET	0.710128	0.767479	0.63491
	AE+ECFP	0.760872	0.769219	0.640393
	BET+ECFP	0.765319	0.767819	0.627158
	AE+BET+ECFP	0.753935	0.775273	0.642065
CHEMBL3785	BET	0.613361	0.670295	0.58161
	AE	0.586961	0.663028	0.472998
	ECFP	0.661229	0.43924	0.616259
	AE+BET	0.608706	0.676099	0.567082
	AE+ECFP	0.656948	0.621186	0.60278
	BET+ECFP	0.661913	0.622934	0.629599
	AE+BET+ECFP	0.649857	0.656479	0.614109
CHEMBL3869	BET	0.514782	0.567652	0.439594
	AE	0.457173	0.587227	0.373426
	ECFP	0.614768	0.500881	0.523561
	AE+BET	0.500598	0.600287	0.418445
	AE+ECFP	0.591158	0.594334	0.479776
	BET+ECFP	0.609682	0.586066	0.505739
	AE+BET+ECFP	0.579892	0.607443	0.475474

Continued on next page

Continued from previous page

Datasets	fingerprint	GBDT	SVM	RF
CHEMBL4029	BET	0.493514	0.475203	0.458381
	AE	0.45553	0.511832	0.443953
	ECFP	0.44059	0.405609	0.486127
	AE+BET	0.483376	0.504724	0.461876
	AE+ECFP	0.503341	0.512617	0.497673
	BET+ECFP	0.513769	0.496837	0.504118
	AE+BET+ECFP	0.512479	0.51823	0.496245
CHEMBL4073	BET	0.652577	0.689511	0.602777
	AE	0.606548	0.678616	0.534193
	ECFP	0.667583	0.659552	0.666022
	AE+BET	0.639375	0.695865	0.585137
	AE+ECFP	0.672873	0.700546	0.641489
	BET+ECFP	0.690432	0.704901	0.668595
	AE+BET+ECFP	0.676001	0.708619	0.641988
CHEMBL4076	BET	0.277668	0.311339	0.214829
	AE	0.279451	0.261675	0.231845
	ECFP	0.225612	0.071203	0.29035
	AE+BET	0.293286	0.308486	0.241167
	AE+ECFP	0.324557	0.276031	0.324508
	BET+ECFP	0.31539	0.294745	0.307768
	AE+BET+ECFP	0.3278	0.319204	0.306306
CHEMBL4223	BET	0.21969	0.173305	0.203011
	AE	0.208654	0.190557	0.191421
	ECFP	0.281022	0.275099	0.263478
	AE+BET	0.230131	0.197337	0.215015
	AE+ECFP	0.293708	0.263082	0.265221
	BET+ECFP	0.289312	0.258988	0.262841
	AE+BET+ECFP	0.282582	0.248741	0.257156
CHEMBL4306	BET	0.61224	0.608568	0.581392
	AE	0.61646	0.601805	0.578618
	ECFP	0.631006	0.607656	0.60371
	AE+BET	0.622592	0.620596	0.591154
	AE+ECFP	0.652086	0.641184	0.608496
	BET+ECFP	0.648038	0.644568	0.611355
	AE+BET+ECFP	0.647906	0.645262	0.608922
CHEMBL4394	BET	0.577518	0.58291	0.551666
	AE	0.574852	0.579621	0.553623
	ECFP	0.591076	0.543297	0.597299
	AE+BET	0.583893	0.592588	0.560804
	AE+ECFP	0.612326	0.595118	0.59692
	BET+ECFP	0.608232	0.593616	0.594947
	AE+BET+ECFP	0.608262	0.602162	0.589789

Continued on next page

Continued from previous page

Datasets	fingerprint	GBDT	SVM	RF
CHEMBL4409	BET	0.61905	0.678518	0.465304
	AE	0.610891	0.691453	0.443212
	ECFP	0.681743	0.62478	0.40081
	AE+BET	0.627221	0.698799	0.46867
	AE+ECFP	0.686082	0.697436	0.463688
	BET+ECFP	0.684288	0.690662	0.463244
	AE+BET+ECFP	0.675409	0.705871	0.474787
CHEMBL4427	BET	0.700236	0.755215	0.655395
	AE	0.684052	0.752106	0.662723
	ECFP	0.743841	0.640065	0.730034
	AE+BET	0.699798	0.764931	0.673586
	AE+ECFP	0.743509	0.732647	0.72426
	BET+ECFP	0.750931	0.731359	0.723983
	AE+BET+ECFP	0.738758	0.75204	0.715463
CHEMBL4462	BET	0.691759	0.7515	0.571664
	AE	0.674902	0.757635	0.565948
	ECFP	0.76924	0.702656	0.671018
	AE+BET	0.694266	0.764834	0.609199
	AE+ECFP	0.761569	0.756159	0.671215
	BET+ECFP	0.761759	0.754334	0.65819
	AE+BET+ECFP	0.748275	0.765502	0.660643
CHEMBL4507	BET	-0.027029	0.093619	-0.039947
	AE	-0.126525	-0.075379	-0.088539
	ECFP	0.03401	-0.015453	-0.100268
	AE+BET	-0.0634	0.01683	-0.051214
	AE+ECFP	0.032944	0.016647	-0.0256
	BET+ECFP	0.079052	0.089438	0.003172
	AE+BET+ECFP	0.034881	0.054319	-0.007313
CHEMBL4607	BET	0.542063	0.562215	0.509072
	AE	0.492612	0.57527	0.430233
	ECFP	0.530294	0.495613	0.536208
	AE+BET	0.533611	0.590107	0.4946
	AE+ECFP	0.56781	0.57979	0.523844
	BET+ECFP	0.573343	0.56609	0.545937
	AE+BET+ECFP	0.570454	0.590095	0.531335
CHEMBL4616	BET	0.501466	0.519542	0.428054
	AE	0.474925	0.517015	0.355614
	ECFP	0.509898	0.491681	0.392987
	AE+BET	0.50373	0.537543	0.408058
	AE+ECFP	0.540573	0.542513	0.396894
	BET+ECFP	0.546194	0.542818	0.428913
	AE+BET+ECFP	0.541745	0.551474	0.417645

Continued on next page

Continued from previous page

Datasets	fingerprint	GBDT	SVM	RF
CHEMBL4691	BET	0.579745	0.569947	0.549988
	AE	0.531038	0.58214	0.524551
	ECFP	0.582329	0.542111	0.588788
	AE+BET	0.566323	0.59071	0.545939
	AE+ECFP	0.605624	0.607874	0.586099
	BET+ECFP	0.620028	0.603638	0.597064
	AE+BET+ECFP	0.60831	0.612743	0.583676
CHEMBL4767	BET	0.468526	0.523028	0.411108
	AE	0.478473	0.548815	0.454028
	ECFP	0.46758	0.418007	0.48034
	AE+BET	0.485878	0.551577	0.449406
	AE+ECFP	0.536794	0.537658	0.513978
	BET+ECFP	0.526986	0.520104	0.487727
	AE+BET+ECFP	0.531562	0.549723	0.495441
CHEMBL4789	BET	0.572719	0.604226	0.517499
	AE	0.503353	0.582607	0.458187
	ECFP	0.514254	0.452584	0.477479
	AE+BET	0.550602	0.604743	0.503639
	AE+ECFP	0.557618	0.569857	0.502389
	BET+ECFP	0.583125	0.577191	0.525537
	AE+BET+ECFP	0.575006	0.596417	0.519233
CHEMBL4835	BET	0.657588	0.694949	0.606898
	AE	0.599752	0.700655	0.56931
	ECFP	0.706406	0.619398	0.635331
	AE+BET	0.643495	0.709526	0.616847
	AE+ECFP	0.688379	0.696117	0.650182
	BET+ECFP	0.706429	0.696884	0.652706
	AE+BET+ECFP	0.687719	0.710567	0.651934
CHEMBL5247	BET	0.267766	0.349296	0.236977
	AE	0.28337	0.374775	0.226504
	ECFP	0.240106	0.118433	0.222214
	AE+BET	0.291131	0.379038	0.251377
	AE+ECFP	0.300608	0.333748	0.261293
	BET+ECFP	0.292693	0.319094	0.263767
	AE+BET+ECFP	0.305165	0.365001	0.268895
CHEMBL5314	BET	0.633217	0.666047	0.58214
	AE	0.587215	0.683692	0.464028
	ECFP	0.644498	0.552545	0.558189
	AE+BET	0.624688	0.688833	0.554262
	AE+ECFP	0.659173	0.66133	0.566579
	BET+ECFP	0.668569	0.650853	0.601592
	AE+BET+ECFP	0.660532	0.677976	0.587264

Continued on next page

Continued from previous page

Datasets	fingerprint	GBDT	SVM	RF
CHEMBL5319	BET	0.343894	0.328837	0.323286
	AE	0.367907	0.355379	0.363169
	ECFP	0.379331	0.384006	0.418384
	AE+BET	0.364424	0.356461	0.353438
	AE+ECFP	0.412675	0.40149	0.424867
	BET+ECFP	0.400117	0.392893	0.411048
	AE+BET+ECFP	0.40197	0.392794	0.405842
CHEMBL5378	BET	0.289793	0.314585	0.271908
	AE	0.185154	0.285106	0.218772
	ECFP	0.351002	0.307685	0.351491
	AE+BET	0.260042	0.319091	0.261176
	AE+ECFP	0.340149	0.335856	0.327489
	BET+ECFP	0.364123	0.351748	0.342759
	AE+BET+ECFP	0.336812	0.346601	0.320392
CHEMBL5398	BET	-0.205906	-0.0805	-0.263867
	AE	-0.113899	0.001201	-0.015012
	ECFP	-0.104489	-0.268647	-0.050278
	AE+BET	-0.140884	-0.034206	-0.099612
	AE+ECFP	0.008158	-0.052477	0.045738
	BET+ECFP	-0.040567	-0.089442	-0.075613
	AE+BET+ECFP	-0.029875	-0.039617	-0.020977
CHEMBL5480	BET	0.317973	0.247712	0.290015
	AE	0.347892	0.271002	0.319906
	ECFP	0.301088	0.27603	0.318043
	AE+BET	0.343936	0.285489	0.313453
	AE+ECFP	0.360074	0.33773	0.344688
	BET+ECFP	0.348497	0.335199	0.333425
	AE+BET+ECFP	0.360342	0.337658	0.337589
CHEMBL5493	BET	0.605171	0.601432	0.581091
	AE	0.613421	0.641046	0.58664
	ECFP	0.607977	0.457724	0.589745
	AE+BET	0.616695	0.634029	0.598859
	AE+ECFP	0.654049	0.611344	0.626361
	BET+ECFP	0.641688	0.586218	0.61173
	AE+BET+ECFP	0.647018	0.625129	0.621147
CHEMBL5918	BET	-0.083952	-0.070302	-0.122371
	AE	-0.065463	-0.076292	0.030573
	ECFP	-0.132382	-0.130215	-0.08565
	AE+BET	-0.064733	-0.064373	-0.024335
	AE+ECFP	-0.049239	-0.062509	0.020341
	BET+ECFP	-0.070004	-0.061291	-0.050605
	AE+BET+ECFP	-0.050456	-0.052876	-0.00455

Continued on next page

Continued from previous page

Datasets	fingerprint	GBDT	SVM	RF
CHEMBL6003	BET	0.141186	0.141531	0.046171
	AE	0.056501	0.106471	0.012516
	ECFP	0.126068	0.012989	0.062401
	AE+BET	0.125391	0.132417	0.061482
	AE+ECFP	0.131756	0.112681	0.07745
	BET+ECFP	0.153133	0.129032	0.086591
	AE+BET+ECFP	0.146374	0.137281	0.086778
CHEMBL6007	BET	0.589407	0.580873	0.571826
	AE	0.518248	0.555003	0.510493
	ECFP	0.563919	0.545703	0.536611
	AE+BET	0.569781	0.577803	0.559908
	AE+ECFP	0.583279	0.586295	0.554538
	BET+ECFP	0.602479	0.597002	0.574198
	AE+BET+ECFP	0.594509	0.595869	0.570627
CHEMBL1250348	BET	0.596897	0.71323	0.426597
	AE	0.604313	0.718534	0.35605
	ECFP	0.699631	0.616595	0.711373
	AE+BET	0.611531	0.726004	0.444985
	AE+ECFP	0.689661	0.700341	0.617926
	BET+ECFP	0.679664	0.69491	0.637094
	AE+BET+ECFP	0.669176	0.715185	0.589556
CHEMBL1293255	BET	0.61166	0.639915	0.573853
	AE	0.602649	0.635655	0.561983
	ECFP	0.596466	0.537045	0.568418
	AE+BET	0.618066	0.649056	0.582628
	AE+ECFP	0.633105	0.624443	0.597263
	BET+ECFP	0.631608	0.625542	0.594872
	AE+BET+ECFP	0.635593	0.642617	0.599422
CHEMBL1293293	BET	0.517217	0.603765	0.470475
	AE	0.52454	0.595831	0.469749
	ECFP	0.588941	0.382333	0.574788
	AE+BET	0.533209	0.61673	0.484432
	AE+ECFP	0.603241	0.553993	0.561897
	BET+ECFP	0.592651	0.55618	0.558944
	AE+BET+ECFP	0.587301	0.591742	0.54512
CHEMBL1741179	BET	0.332699	0.340651	0.322327
	AE	0.317407	0.365011	0.308864
	ECFP	0.266121	0.144351	0.26598
	AE+BET	0.338358	0.370757	0.32856
	AE+ECFP	0.326775	0.311537	0.309995
	BET+ECFP	0.333219	0.297393	0.312246
	AE+BET+ECFP	0.341909	0.34097	0.322893

Continued on next page

Continued from previous page

Datasets	fingerprint	GBDT	SVM	RF
CHEMBL1741186	BET	0.52169	0.576315	0.416863
	AE	0.491112	0.581535	0.41261
	ECFP	0.543509	0.49723	0.387228
	AE+BET	0.520304	0.595922	0.427987
	AE+ECFP	0.561898	0.584056	0.428851
	BET+ECFP	0.567861	0.579407	0.425595
	AE+BET+ECFP	0.560438	0.598051	0.434782
CHEMBL1741200	BET	0.105829	0.05879	0.077001
	AE	0.142312	0.106794	0.107664
	ECFP	0.064602	-0.054189	0.058969
	AE+BET	0.133814	0.09736	0.099733
	AE+ECFP	0.143117	0.081669	0.104761
	BET+ECFP	0.122487	0.059007	0.085121
	AE+BET+ECFP	0.14277	0.093417	0.101647
CHEMBL1741213	BET	0.118502	0.068347	0.1136
	AE	0.12111	0.062766	0.1079
	ECFP	0.069961	0.042552	0.117241
	AE+BET	0.133106	0.094925	0.118617
	AE+ECFP	0.143193	0.103969	0.128223
	BET+ECFP	0.143425	0.109988	0.13018
	AE+BET+ECFP	0.152147	0.117985	0.129926
CHEMBL2424504	BET	0.582534	0.614243	0.531371
	AE	0.56536	0.603731	0.536194
	ECFP	0.615359	0.572091	0.60404
	AE+BET	0.584548	0.620777	0.554479
	AE+ECFP	0.619941	0.617602	0.600976
	BET+ECFP	0.622198	0.62638	0.59606
	AE+BET+ECFP	0.615944	0.629886	0.592717
CHEMBL3392948	BET	0.46476	0.496521	0.403373
	AE	0.449825	0.492082	0.413967
	ECFP	0.431168	0.358633	0.451634
	AE+BET	0.465916	0.51153	0.419126
	AE+ECFP	0.490702	0.493092	0.472761
	BET+ECFP	0.495643	0.493883	0.464058
	AE+BET+ECFP	0.495921	0.516309	0.461645
CHEMBL3714079	BET	0.519496	0.526836	0.47723
	AE	0.477735	0.522232	0.466729
	ECFP	0.448519	0.378059	0.443486
	AE+BET	0.509783	0.541665	0.489555
	AE+ECFP	0.514916	0.515389	0.485122
	BET+ECFP	0.523938	0.519454	0.485165
	AE+BET+ECFP	0.527644	0.542101	0.494658

S5 Docking details

In this study, we used AutoDock Vina software to dock estradiol cypionate, bosentan, givinostat with the GPR84 protein (PDB ID: 2HDA), and trimebutine, verapamil, gefitinib with the F2RL3 protein (PDB ID: 2ZPK). The results of nine conformations generated by docking are shown in Tables S7, S8, S9, S10, S11, and S12.

Table S7: Details of the docking between estradiol cypionate and GPR84 (PDB ID: 2HDA)

mode	affinity (kcal/mol)	dist from rmsd 1.b.	best mode rmsd u.b.
1	-8.2	0.000	0.000
2	-8.0	1.612	2.778
3	-7.5	16.434	18.151
4	-7.1	2.089	2.699
5	-6.9	16.290	18.765
6	-6.5	15.720	17.866
7	-6.4	15.877	18.440
8	-6.1	16.291	18.212
9	-5.1	16.055	18.358

Table S8: Details of the docking between bosentan and GPR84 (PDB ID: 2HDA)

mode	affinity (kcal/mol)	dist from rmsd 1.b.	best mode rmsd u.b.
1	-8.3	0.000	0.000
2	-7.8	2.264	3.337
3	-7.5	6.297	11.375
4	-7.4	9.352	13.764
5	-7.3	3.114	5.271
6	-7.3	1.691	2.533
7	-7.3	13.630	15.662
8	-7.2	9.884	13.490
9	-7.2	8.314	12.538

Table S9: Details of the docking between givinostat and GPR84 (PDB ID: 2HDA)

mode	affinity (kcal/mol)	dist from rmsd 1.b.	best mode rmsd u.b.
1	-9.1	0.000	0.000
2	-8.9	5.020	12.892
3	-8.7	4.171	7.594
4	-8.3	5.499	12.887
5	-8.1	29.790	34.188
6	-8.1	26.342	29.448
7	-8.1	27.016	29.512
8	-8.0	30.844	35.569
9	-8.0	30.646	33.461

S6 Binding Affinity Prediction Results

We used the constructed machine learning model to predict the binding affinity of 6001 drugs in DrugBank. Due to space limitations, the complete prediction results can be obtained for free on GitHub (https://github.com/hahaha3758/Drug_repurposing).

Table S10: Details of the docking between trimebutine and F2RL3 (PDB ID: 2ZPK)

mode	affinity (kcal/mol)	dist from rmsd 1.b.	best mode rmsd u.b.
1	-5.0	0.000	0.000
2	-5.0	0.282	2.594
3	-4.9	4.116	7.253
4	-4.9	3.061	6.101
5	-4.8	1.628	3.393
6	-4.7	2.806	5.188
7	-4.7	3.102	5.564
8	-4.7	2.563	5.348
9	-4.7	3.044	6.603

Table S11: Details of the docking between verapamil and F2RL3 (PDB ID: 2ZPK)

mode	affinity (kcal/mol)	dist from rmsd 1.b.	best mode rmsd u.b.
1	-6.1	0.000	0.000
2	-6.0	29.605	34.369
3	-5.9	25.143	28.769
4	-5.9	24.222	27.896
5	-5.9	25.727	28.753
6	-5.8	25.577	28.198
7	-5.7	24.420	28.218
8	-5.7	24.135	28.094
9	-5.6	25.658	27.958

Table S12: Details of the docking between gefitinib and F2RL3 (PDB ID: 2ZPK)

mode	affinity (kcal/mol)	dist from rmsd 1.b.	best mode rmsd u.b.
1	-6.6	0.000	0.000
2	-6.6	2.962	5.921
3	-6.6	7.652	10.135
4	-6.3	23.388	26.360
5	-6.2	2.461	3.536
6	-6.1	23.078	26.052
7	-6.1	3.430	4.249
8	-6.1	2.744	4.286
9	-6.0	21.044	24.423
